# Plasma lipidomics profile in pregnancy and gestational diabetes risk: a prospective study in a multiracial/ethnic cohort

**DOI:** 10.1136/bmjdrc-2020-001551

**Published:** 2021-03-05

**Authors:** Mohammad L Rahman, Yen-Chen A Feng, Oliver Fiehn, Paul S Albert, Michael Y Tsai, Yeyi Zhu, Xiaobin Wang, Fasil Tekola-Ayele, Liming Liang, Cuilin Zhang

**Affiliations:** 1 Department of Population Medicine and Harvard Pilgrim Health Care Institute, Harvard Medical School, Boston, Massachusetts, USA; 2 Epidemiology Branch, Division of Intramural Population Health Research, Eunice Kennedy Shriver National Institute of Child Health and Human Development, Bethesda, Maryland, USA; 3 Massachusetts General Hospital Center for Genomic Medicine, Boston, Massachusetts, USA; 4 Program in Medical and Population Genetics, Broad Institute Harvard, Cambridge, Massachusetts, USA; 5 West Coast Metabolomics Center, University of California Davis, Davis, California, USA; 6 Division of Cancer Epidemiology and Genetics, National Cancer Institute, Bethesda, Maryland, USA; 7 Laboratory Medicine and Pathology, University of Minnesota System, Minneapolis, Minnesota, USA; 8 Division of Research, Kaiser Permanente Northern California, Oakland, California, USA; 9 Department of Population, Family, and Reproductive Health, Johns Hopkins University Bloomberg School of Public Health, Baltimore, Maryland, USA; 10 Department of Biostatistics, Harvard University T H Chan School of Public Health, Boston, Massachusetts, USA

**Keywords:** diabetes, gestational, pregnancy, lipids, metabolism

## Abstract

**Introduction:**

Disruption of lipid metabolism is implicated in gestational diabetes (GDM). However, prospective studies on lipidomics and GDM risk in race/ethnically diverse populations are sparse. Here, we aimed to (1) identify lipid networks in early pregnancy to mid-pregnancy that are associated with subsequent GDM risk and (2) examine the associations of lipid networks with glycemic biomarkers to understand the underlying mechanisms.

**Research design and methods:**

This study included 107 GDM cases confirmed using the Carpenter and Coustan criteria and 214 non-GDM matched controls from the National Institute of Child Health and Human Development Fetal Growth Studies-Singleton cohort, untargeted lipidomics data of 420 metabolites (328 annotated and 92 unannotated), and information on glycemic biomarkers in maternal plasma at visit 0 (10–14 weeks) and visit 1 (15–26 weeks). We constructed lipid networks using weighted correlation network analysis technique. We examined prospective associations of lipid networks and individual lipids with GDM risk using linear mixed effect models. Furthermore, we calculated Pearson’s partial correlation for GDM-related lipid networks and individual lipids with plasma glucose, insulin, C-peptide and glycated hemoglobin at both study visits.

**Results:**

Lipid networks primarily characterized by elevated plasma diglycerides and short, saturated/low unsaturated triglycerides and lower plasma cholesteryl esters, sphingomyelins and phosphatidylcholines were associated with higher risk of developing GDM (false discovery rate (FDR) <0.05). Among individual lipids, 58 metabolites at visit 0 and 96 metabolites at visit 1 (40 metabolites at both time points) significantly differed between women who developed GDM and who did not (FDR <0.05). Furthermore, GDM-related lipid networks and individual lipids showed consistent correlations with maternal glycemic markers particularly in early pregnancy at visit 0.

**Conclusions:**

Plasma lipid metabolites in early pregnancy both individually and interactively in distinct networks were associated with subsequent GDM risk in race/ethnically diverse US women. Future research is warranted to assess lipid metabolites as etiologic markers of GDM.

**Significance of this study**

**What is already known about this subject?**

- Emerging epidemiological studies in non-pregnant population have identified lipid metabolites prospectively associated with insulin resistance and type 2 diabetes; however, prospective studies among pregnant women on lipidomic profiles and gestational diabetes (GDM) are sparse.
- We examined plasma lipidomic profile in early pregnancy to mid-pregnancy in relation to the subsequent risk of GDM in a multiracial/ethnic US population.

**What are the new findings?**

- We identified a list of glycerolipids and some glycerophospholipids, sterol lipids and sphingomyelins measured in maternal plasma as early as in 10–14 weeks of gestation individually and interactively in distinct metabolite networks were associated with GDM risk.
- These metabolites and their interactive networks also showed correlation patterns with maternal non-fasting and fasting glycemic biomarkers that were in consistent with findings on GDM.

**How might these results change the focus of research or clinical practice?**

- This study sheds light on the importance of low-molecular-weight intermediate lipid metabolites as etiological biomarkers of GDM.

## Introduction

Gestational diabetes (GDM) is one of the most common pregnancy complications and has been linked with both short-term and long-term adverse health consequences for the woman and her child. For instance, women diagnosed with GDM are at 4.8–11.5 times higher risk of developing type 2 diabetes later in life.^1^ Their children are more likely to be macrosomic at birth and are at a higher risk of developing childhood obesity and glucose intolerance during adulthood.^2 3^ Therefore, identifying etiological biomarkers and modifiable risk factors of GDM are important milestones to design effective intervention strategy and improve the health and well-being of both women and their children.^4^

Prior studies point to disturbances in lipid metabolism in developing glucose homeostasis,^5^ however the traditional lipid biomarkers such as total triglycerides and cholesterol do not reflect the complexity of the altered lipid metabolism associated with GDM. Lipidomics is an emerging tool of quantitative analysis of the full spectra of low-molecular-weight intermediate lipid metabolites, which may reflect a snapshot of the dynamic biochemical activities and provide new insights into the underlying etiology of GDM.^6 7^ A longitudinal evaluation of the lipidome in early pregnancy to mid-pregnancy can also capture the temporal variation in lipid profiles, accounting for exogenous (diet) and endogenous lipid metabolism.

Emerging studies among non-pregnant individuals have identified novel lipid biomarkers prospectively associated with insulin resistance,^8^ type 2 diabetes^9^ and transition to type 2 diabetes from GDM.^10 11^ Only two studies have prospectively evaluated the lipidome with GDM risk.^12 13^ However, those studies were conducted exclusively among white Caucasian or Han Chinese populations, limiting the generalisability of findings to other populations. Inference from these studies were also hindered by small sample size (GDM cases=21)^12^ or liability for residual confounding because of a lack of information on conventional risk factors, such as family history of diabetes.^13^ To address these critical research gaps, we aimed to (1) prospectively evaluate the associations of maternal plasma lipidomics profile at two-time windows before GDM diagnosis (ie, in early pregnancy and mid-pregnancy) with the subsequent risk of developing GDM in a race/ethnically diverse US population. We also aimed to examine the associations of lipidomics profile with maternal glycemic biomarkers to understand the etiologic role of lipid metabolites in the development of GDM.

## Subjects and methods

### Study population and design

We conducted a case-control study nested within the *Eunice Kennedy Shriver* National Institute of Child Health and Human Development (NICHD) Fetal Growth Studies-Singletons cohort, which is a multicenter, multiracial/ethnic prospective pregnancy cohort. During 2009–2013, 2802 pregnant women aged 18–40 years without pre-existing hypertension, diabetes, cancer and other chronic diseases were recruited between 8 and 13 weeks of pregnancy through 12 clinical centers across the USA. The detail of the study has been previously described.^14^

On recruitment at 8–13 weeks (visit 0), women were scheduled to attend five in-hospital follow-up visits at targeted gestational weeks: 16–22 (visit 1), 24–29 (visit 2), 30–33 (visit 3), 34–37 (visit 4) and 38–41 (visit 5). Venous blood samples were collected at visits 0, 1, 2 and 4, where only visit 1 samples were collected after an overnight fast. Because some women arrived late for their scheduled visit, the actual blood collection windows ranged slightly beyond the targeted time windows (ie, weeks 10–14 (visit 0), 15–26 (visit 1), 23–31 (visit 2), 33–39 (visit 4)), the first two of which were before GDM diagnosis. Plasma samples were processed immediately after blood collection and stored at −80°C until analysis.

Within the cohort, we identified 107 incident GDM cases based on the Carpenter and Coustan criteria through medical records review of oral glucose tolerance test (OGTT) results following the American College of Obstetrics and Gynecologists recommendations (online supplemental figure S1).^15 16^ OGTT was performed at a mean (±SD) gestational age of 27.5 (±4.3) weeks. We also selected 214 controls matched with the cases in a 2:1 ratio on age (±2 years), self-reported race/ethnicity and gestational week of blood collection (±2 weeks). Of note, the majority of controls (n=195) were screened for GDM using the 50 g glucose challenge test. For those without routine GDM screening (n=19), 12 went through an OGTT with normal glucose values below the Carpenter and Coustan criteria thresholds and the remaining were free of hospital discharge diagnosis of GDM.

10.1136/bmjdrc-2020-001551.supp1 Supplementary data

10.1136/bmjdrc-2020-001551.supp2 Supplementary data

### Laboratory assays

Lipidome analysis was performed at the West Coast Metabolomics Center, University of California Davis Genome Center. Details of laboratory methods used to quantify lipids were presented elsewhere.^17^ Briefly, plasma lipidome was quantified using high-throughput liquid chromatography quadruple time-of-flight mass spectrometry (LC-QTOF MS/MS).^18 19^ Internal standards were used for the calibration of retention times.^17^ Signals across samples were corrected by a bioreclamation quality control plasma-based normalization method.

A total of 420 non-targeted lipid metabolites, including 328 annotated and 92 unannotated metabolites were profiled at visits 0, 1, 2 and 4. The annotated metabolites belonged to four major lipid categories: glycerolipids (n=145), including monoglycerides (MG, n=3), diglycerides (DG, n=16), triglycerides (TG, n=126); glycerophospholipids (n=116), including phosphatidylcholines (PC, n=83), phosphatidylethanolamines (PE, n=11) and lysophosphatidylcholines (LPC, n=22); sphingolipids (n=62), including sphingomyelins (SM, n=34), ceramides (Cer, n=15), glucosylceramides (GlcCer, n=9), lactosylceramides (LacCer, n=4) and sterol lipids (n=20), primarily cholesteryl esters (CE, n=18).

We also measured a panel of glycemic biomarkers in maternal plasma at the same study visits. Concentrations of glucose, insulin and C-peptide were measured using hexokinase, immunosorbent, sandwich immunoassay and immunoturbidimetric assays (Roche Diagnostics, Indianapolis, Indiana), respectively. Glycated hemoglobin (HbA1c) was measured in whole blood using chromatography (Tosoh Bioscience, California and Tokyo, Japan). All assays had inter-assay and intra-assay coefficients of variation <10% and were performed without the knowledge of GDM status.

### Covariates

Data on maternal demographic, lifestyle and clinical factors were collected at each visit using a standardized and structured questionnaire. Potential confounders of exposure-outcome relationship were selected a priori based on causal diagram,^20^ which included maternal age (continuous), family history of diabetes (yes, no), enrollment body mass index (BMI) (continuous), alcohol use before pregnancy (yes, no), race/ethnicity (white, non-Hispanic black, Hispanic and Asians) and gestational age at blood collection (continuous). We also collected information on GDM treatment (intervention using medication, intervention using diet or lifestyle modification but no medication and no intervention undertaken) from medical records. In this low-risk population, women without obesity who smoked 6 months preceding the index pregnancy were ineligible, and only five women with obesity reported smoking during the same period before pregnancy. Thus, in our main analysis, we did not include smoking as a covariate.

### Statistical analysis

All analyses were conducted in R V.3.5.2 (Austria, Vienna) and SAS V.9.4 (SAS Institute, Cary, North Carolina, USA). The bivariate characteristics of GDM cases and non-GDM controls at baseline were compared using linear mixed models, accounting for matched case-control pairs. For associations with GDM risk, lipidome data collected prior to GDM diagnosis at visit 0 (10–14 weeks) and visit 1 (15–26 weeks) were used in the analysis to preserve the temporal relation.

#### Metabolite coregulating network analysis

We applied weighted correlation network analysis (R package) to construct networks of highly correlated lipid metabolites, and examined how metabolites within an interconnecting network collectively influence GDM risk.^21^ Lipidomics data were normalized using quantile normalization to reduce batch effects and then standardized using inverse-normal transformation to remove the effect of potential outliers. To construct metabolite networks, first we obtained network adjacency matrix,^22^ a measure of connectedness between each pair of metabolites, estimated by Pearson’s correlation coefficients. We then transformed network adjacency matrix to topological overlap matrix (TOM),^23^ a measure of connectedness between each pair of metabolites in a network considering their relation to all other metabolites within that network and subsequently performed hierarchical clustering on TOM-based dissimilarity. Consecutively, we applied the Dynamic Tree Cut function^24^ to identify network clusters consisting of highly correlated metabolites that follow a scale-free topology, which is a type of network configuration characterized by a few metabolites having many connections with neighboring metabolites but most metabolites having just a handful of connections.^25^ This analytical approach was adopted based on reports suggesting that most metabolic networks in a biological system follow a scale-free topology.^25^

To evaluate associations between lipid networks and GDM risk, we used the first eigenvector of each identified network in linear mixed-effect models, where metabolite network score was used as the response variable and GDM status as the independent variable, adjusting for age, gestational week at blood collection, enrollment BMI, self-reported race/ethnicity, alcohol use before pregnancy and family history of diabetes. Case-control pair ID was modeled as random intercept to account for correlation within matched pair. Multiple testing was corrected using false discovery rate (FDR). To identify individual metabolites within a network that were driving the associations, we estimated network membership score, a measure of importance of member metabolites within each network, as the correlation between metabolite concentrations and the first eigenvector of respective metabolite network.

Next, we examined correlations between metabolite networks and maternal glycemic markers using Pearson’s partial correlations adjusting for covariates. Participants missing information on insulin (n=1), glucose (n=6) and HbA1c (n=6) at visit 0 and fasting insulin, (n=2), fasting C-peptide (n=2), fasting glucose (n=2) and fasting HbA1c (n=2) at visit 1 were excluded from the analyses. Concentration of glycemic markers was natural log transformed prior to analyses. We applied inverse probability weighting to account for the matched case-control design.

#### Individual metabolite analysis

We also examined individual metabolites, one at a time, in relation to GDM using similar mixed effect models, adjusting for covariates. First, we normalized the data using inverse-normal transformation within batches. We analyzed the data separately by batch and then combined results using inverse-variance weighted meta-analysis to minimize batch effect. Analyses were corrected for multiple testing using FDR. We visualized results in a two-dimensional space by the number of acyl chain carbon atoms (x-axis) and double bond contents (y-axis) of individual metabolites within each lipid class because prior studies reported that the number of acyl carbons and double bonds were important characteristics to determine glycemic risk of endogenous metabolites.^26^ Plots were visually inspected for patterns of association by acyl carbon chain length and double bond contents.

Next, we estimated correlations between individual metabolites with maternal glycemic biomarkers using Pearson’s partial correlation, adjusting for covariates. Finally, leveraging on repetitive measures of lipidomics data, we examined the mean abundance of selected metabolites between GDM cases and controls across pregnancy to assess temporal trend of selected metabolites with GDM status. The case-control difference in metabolite concentrations was assessed using the two-sample t-tests. SEs of the mean and p values were also plotted at each specific time point.

#### Sensitivity analysis

For associations between lipid networks and GDM risk, we conducted sensitivity analysis additionally adjusting for maternal smoking status prior to pregnancy to assess residual confounding of our results by maternal smoking status. To examine potential reverse causation by GDM severity status, we conducted a sensitivity analysis stratified by GDM treatment group (intervention using medication; intervention using diet or lifestyle modification but no medication and no intervention undertaken) at both study visits.

## Results

Women who developed GDM (n=107) were more likely to have higher BMI at the time of enrollment and a positive family history of diabetes compared with non-GDM controls (n=214) (table 1). GDM women also had higher concentrations of fasting glucose, fasting insulin, C-peptide and HbA1c at visit 0.

**Table 1:** Participant characteristics among women with gestational diabetes (GDM) and their matched* controls: the NICHD Fetal Growth Studies-Singleton Cohort

Characteristics	N	GDM cases (n=107)	Non-GDM controls (n=214)	P value
Age, years	321	30.4±5.7	30.5±5.4	
Race/Ethnicity	321			
Non-Hispanic white		25 (23.4%)	50 (23.4%)	
Non-Hispanic black		15 (14.0%)	30 (14.0%)	
Hispanic		41 (38.3%)	82 (38.3%)	
Asian/Pacific Islander		26 (24.3%)	52 (24.3%)	
Enrollment BMI, kg/m^2^	321	27.8±6.2	25.6±5.3	0.0005
Family history of diabetes	321	40 (37.4%)	48 (22.4%)	0.003
Alcohol use before pregnancy	321	61 (57.0%)	137 (64.0%)	0.22
Smoking before pregnancy	321	4 (3.7%)	1 (0.5%)	0.02
Fasting glucose†, mg/dL	313	91.92±14.6	83.38±7.9	<0.001
Fasting insulin†, pmol/L	313	135.41±204.6	66.87±95.2	<0.001
Fasting C-peptide†, nmol/L	313	0.92±0.7	0.60±0.4	<0.001
Fasting HbA1c†, %	313	5.18±0.5	4.95±0.3	0.002

Values are n (%) or mean±SD.

P values were not shown for matching variables: age and race/ethnicity.

*Matching factors: age (±2 years), race/ethnicity (non-Hispanic white, non-Hispanic black, Hispanic or Asian/Pacific Islander) and the gestational week of blood collection (±2 weeks).

†Glycemic biomarkers were measured in fasting samples at 15–26 weeks (visit 1). Sample size for fasting glucose was 98 cases and 213 controls and for fasting insulin, C-peptide and HbA1c was 99 cases and 212 controls. Values were log transformed before fitting into linear mixed effect models.

BMI, body mass index; HbA1c, glycated hemoglobin; NICHD, National Institute of Child Health and Human Development.

### Lipid coregulating networks and GDM risk

**Figure 1:**
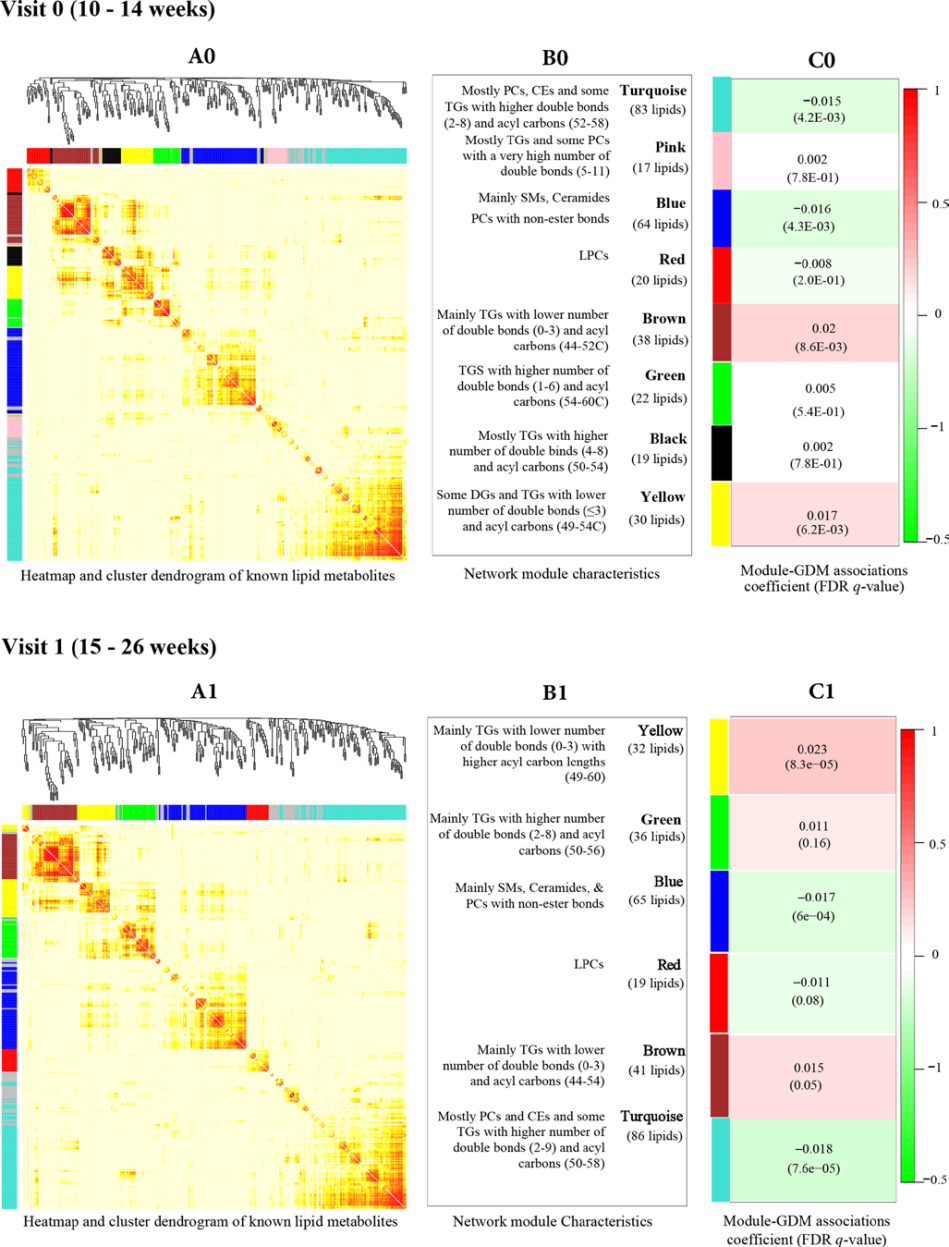
Lipid networks represented by correlated metabolites, as shown in cluster dendrogram and heatmap at visit 0 (A0) and visit 1 (A1). We constructed eight lipid networks at visit 0 (B0) and six networks at visit 1 (B1) from 328 annotated lipid metabolites using weighted correlation network analysis algorithm. Constituents of each lipid network are presented. The associations between lipid networks and GDM risk (coefficient (FDR)) using linear mixed effect models, adjusted for maternal age, enrollment BMI, family history of diabetes, alcohol use before pregnancy, race/ethnicity and gestational age at blood collection at visit 0 (C0) and visit 1 (C1) are presented. BMI, body mass index; CE, cholesteryl ester; FDR, false discovery rate; GDM, gestational diabetes; LPC, lysophosphatidylcholine; PC, phosphatidylcholine; SM, sphingomyelin; TG, triglyceride.

We identified eight coregulating lipid networks at visit 0 and six networks at visit 1; the networks were in general well preserved between study visits (figure 1). In both visits, the ‘yellow’ and ‘brown’ networks, which primarily comprised short, saturated/low unsaturated TGs were positively associated with GDM risk, whereas the ‘turquoise’ network primarily comprised CEs, PCs and some long, polyunsaturated TGs, and the ‘blue’ network primarily comprised SMs and ceramides were negatively associated with GDM risk (FDR <0.05).

### Lipid coregulating networks and glycemic biomarkers

**Figure 2:**
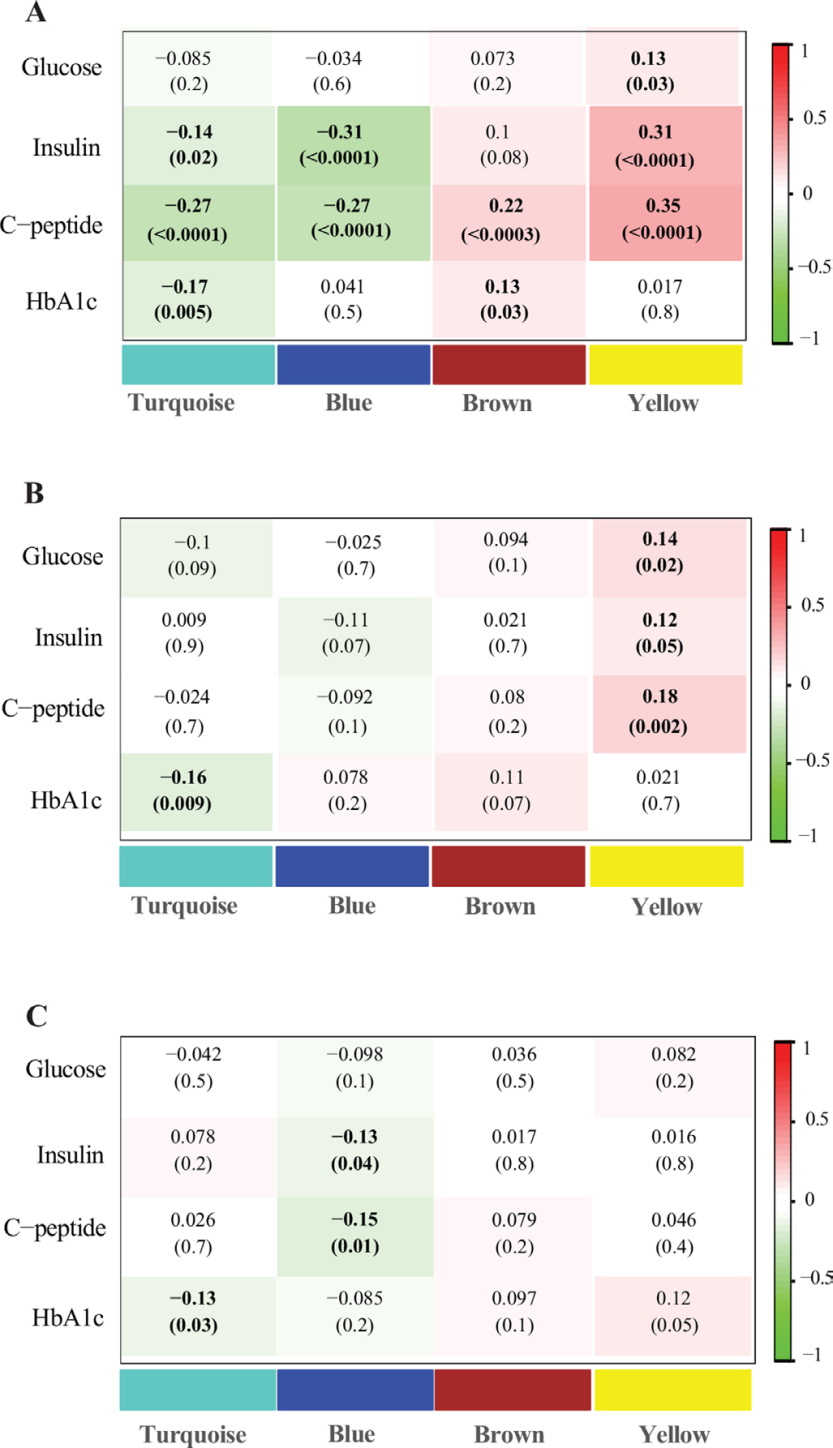
Pearson’s partial correlation coefficients (p values) between GDM-related lipid networks and maternal glycemic markers, adjusted for maternal age, enrollment BMI, family history of diabetes, alcohol use before pregnancy, race/ethnicity and gestational age at blood collection. (A) Cross-sectional associations of lipid networks with non-fasting glycemic markers at visit 0; (B) prospective associations of lipid networks at visit 0 with fasting glycemic markers at visit 1; (C) cross-sectional associations of lipid networks and fasting glycemic markers at visit 1. BMI, body mass index; GDM, gestational diabetes; HbA1c, glycated hemoglobin.

GDM-related lipid networks showed moderate to weak but largely consistent correlations with glycemic biomarkers, although the strength of association differed by study visits (figure 2). In early pregnancy at visit 0, cross-sectional analyses showed that the ‘turquoise’ and ‘blue’ networks were negatively correlated with non-fasting plasma insulin, C-peptide and HbA1c, whereas the ‘brown’ and ‘yellow’ networks showed the opposite pattern. Similar but weaker correlation pattern was observed in prospective analyses of metabolite networks at visit 0 with fasting glycemic markers measured at visit 1. Specifically, the ‘turquoise’ network at visit 0 was negatively correlated with HbA1c (r*=*−0.16, p=0.009) at visit 1, whereas the ‘yellow’ network at visit 0 was positively correlated with fasting glucose (r*=*0.14, p=0.02), insulin (r*=*0.12, p=0.05) and C-peptide (r*=*0.18, p=0.002) at visit 1. Cross-sectional correlations between GDM-related lipid networks and fasting glycemic biomarkers at visit 1 were weaker but showed a consistent pattern.

### Individual lipid metabolites with GDM risk

Table 2 presents associations of selected individual metabolites with GDM risk by study visit (complete results are presented in online supplemental table 1). Specifically, 58 metabolites (47 annotated, 11 unannotated) at visit 0 and 96 metabolites (75 annotated and 21 unannotated) at visit 1 (40 metabolites at both study visits) were significantly associated with GDM risk (FDR <0.05). At visit 0, higher plasma concentrations of 41 metabolites (6 DGs, 30 TGs, 4 PCs and 1 PE) and lower concentration of 6 metabolites (all CEs) were associated with higher risk of GDM. For instance, TG (50:1; 10.80_850.79), a member of the ‘brown’ network, showed the most significant positive association with GDM (β=0.54; FDR=0.0003) at visit 0, whereas CE (18:2; 10.37_671.57), a member of the ‘turquoise’ network showed the most significant negative association (β=−0.52; FDR=0.001). Similar associations were observed at visit 1. For instance, higher plasma concentrations of 64 metabolites (6 GDs, 52 TGs, 3 PEs, 1 PC and 2 SMs) and lower concentrations of 14 metabolites (6 CEs, 4 PCs, 1 LPC and 3 SMs) at visit 1 were associated with higher risk of GDM. Again, TG (50:1; 10.80_850.79) showed the most significant positive association with GDM (β=0.51, FDR=0.0006) at visit 1, whereas CE (18:1; 10.85_673.59), a member of the ‘turquoise’ network, showed the most significant negative association (β=−0.53, FDR=0.004).

10.1136/bmjdrc-2020-001551.supp3 Supplementary data

**Table 2:** Selected individual metabolites and their associations with GDM risk by study visit

Metabolite	RT_m/z	Corresponding lipid network	Visit 0 (10–14 weeks)			Visit 1 (15–26 weeks)		
			Network membership score*	Estimate	FDR	Network membership score*	Estimate	FDR
TG (50:1)	10.80_850.79	Brown	0.80	0.54	0.0003	0.80	0.51	0.0006
TG (50:2)	10.41_853.73	Brown	0.55	0.55	0.0003	0.64	0.43	0.0037
TG (50:2)	10.41_848.77	Brown	0.73	0.54	0.0003	0.73	0.43	0.0037
TG (54:5)	10.32_903.74			0.50	0.0011		0.44	0.0043
CE (18:2)	10.37_671.57	Turquoise	0.95	−0.52	0.0011	0.94	−0.33	0.0290
Unannotated	10.80_551.50			0.50	0.0011		0.51	0.0006
TG (48:1)	10.38_822.75	Brown	0.93	0.48	0.0016	0.92	0.42	0.0049
Unannotated	10.39_549.49			0.47	0.0016		0.37	0.0097
TG (48:1)	10.38_827.71	Brown	0.93	0.48	0.0017	0.94	0.36	0.0170
TG (50:1)	10.80_855.74	Brown	0.51	0.47	0.0029	0.53	0.41	0.0059
DG (34:1)	6.85_612.56	Yellow	0.83	0.44	0.0048	0.75	0.45	0.0031
TG (52:1)	11.20_878.82	Yellow	0.81	0.43	0.0054	0.80	0.50	0.0011
PC (38:3)	5.79_812.61			0.42	0.0054		0.36	0.0200
Unannotated	10.80_577.52			0.42	0.0054		0.44	0.0021
TG (48:2)	9.98_820.74	Brown	0.84	0.43	0.0054	0.88	0.34	0.0230
TG (50:3)	10.01_846.76	Yellow	0.80	0.42	0.0054	0.76	0.41	0.0046
TG (52:1)	11.20_883.77	Brown	0.57	0.42	0.0059	0.63	0.48	0.0016
TG (58:6)	10.65_957.79			0.44	0.0059		0.43	0.0047
DG (34:1)	6.85_617.51	Yellow	0.67	0.42	0.0059	0.46	0.43	0.0037
Unannotated	11.20_899.75			0.40	0.0064		0.40	0.0047
TG (48:0)	10.81_824.77	Brown	0.78	0.41	0.0072	0.82	0.52	0.0008
TG (58:6)	10.65_952.83	Yellow	0.68	0.41	0.0117	0.71	0.44	0.0037
CE (18:1)	10.85_673.59	Turquoise	0.89	−0.40	0.0133	0.88	−0.53	0.0004
TG (46:0)	10.36_796.74	Brown	0.93	0.37	0.0165	0.96	0.41	0.0073
Unannotated	6.39_615.49			0.36	0.0197		0.47	0.0016
Unannotated	10.31_951.81			0.37	0.0204		0.39	0.0071
TG (51:1)	10.93_869.76	Yellow	0.80	0.37	0.0221	0.76	0.36	0.0120
TG (51:1)	10.93_864.80	Yellow	0.90	0.36	0.0221	0.84	0.36	0.0120
CE (18:1)	10.85_668.63	Turquoise	0.94	−0.36	0.0267	0.92	−0.40	0.0047
TG (54:2)	11.20_904.83	Yellow	0.79	0.36	0.0281	0.82	0.43	0.0047
TG (54:1)	11.56_906.85	Yellow	0.77	0.35	0.0328	0.86	0.45	0.0042
CE (22:6)	9.88_719.57	Turquoise	0.94	−0.34	0.0328	0.94	−0.34	0.0250
TG (56:4)	10.88_933.79			0.33	0.0388		0.40	0.0049
TG (49:1)	10.59_836.77	Yellow	0.80	0.32	0.0415	0.74	0.28	0.0470
Unannotated	10.17_896.79			0.32	0.0415		0.33	0.0270
TG (48:0)	10.81_829.73	Brown	0.67	0.32	0.0484	0.73	0.41	0.0071

FDR q values were calculated based on 420 individual tests for 420 metabolites.

*Network membership score represents a measure of importance for member metabolites within a network and ranges between −1 and 1. Network membership scores were not presented for metabolites that failed to form a network and for unannotated metabolites.

CE, cholesteryl esters; DG, diglyceride; FDR, false discovery rate; GDM, gestational diabetes; GlcCer, glucosylceramide; LPC, lysophosphatidylcholine; PC, phosphatidylcholine; RT_m/z, retention time_mass-to-charge ratio; SM, sphingomyelin; TG, triglyceride.

### Individual lipid metabolite-GDM relations by acyl carbon chain length and bond characteristics

We illustrated associations of individual lipid metabolites with GDM risk by acyl carbon chain length and double bond contents of the metabolites (online supplemental figure S2), as prior studies have identified these properties as important determinants of cardiometabolic risk.^8 27^ Visual inspection of scatter plots suggests that short, saturated or less unsaturated TGs and saturated SMs were more likely to be associated with higher risk of GDM. No clear association pattern was observed for other lipid classes.

10.1136/bmjdrc-2020-001551.supp4 Supplementary data

### GDM-related individual lipid metabolites and glycemic biomarkers

**Figure 3:**
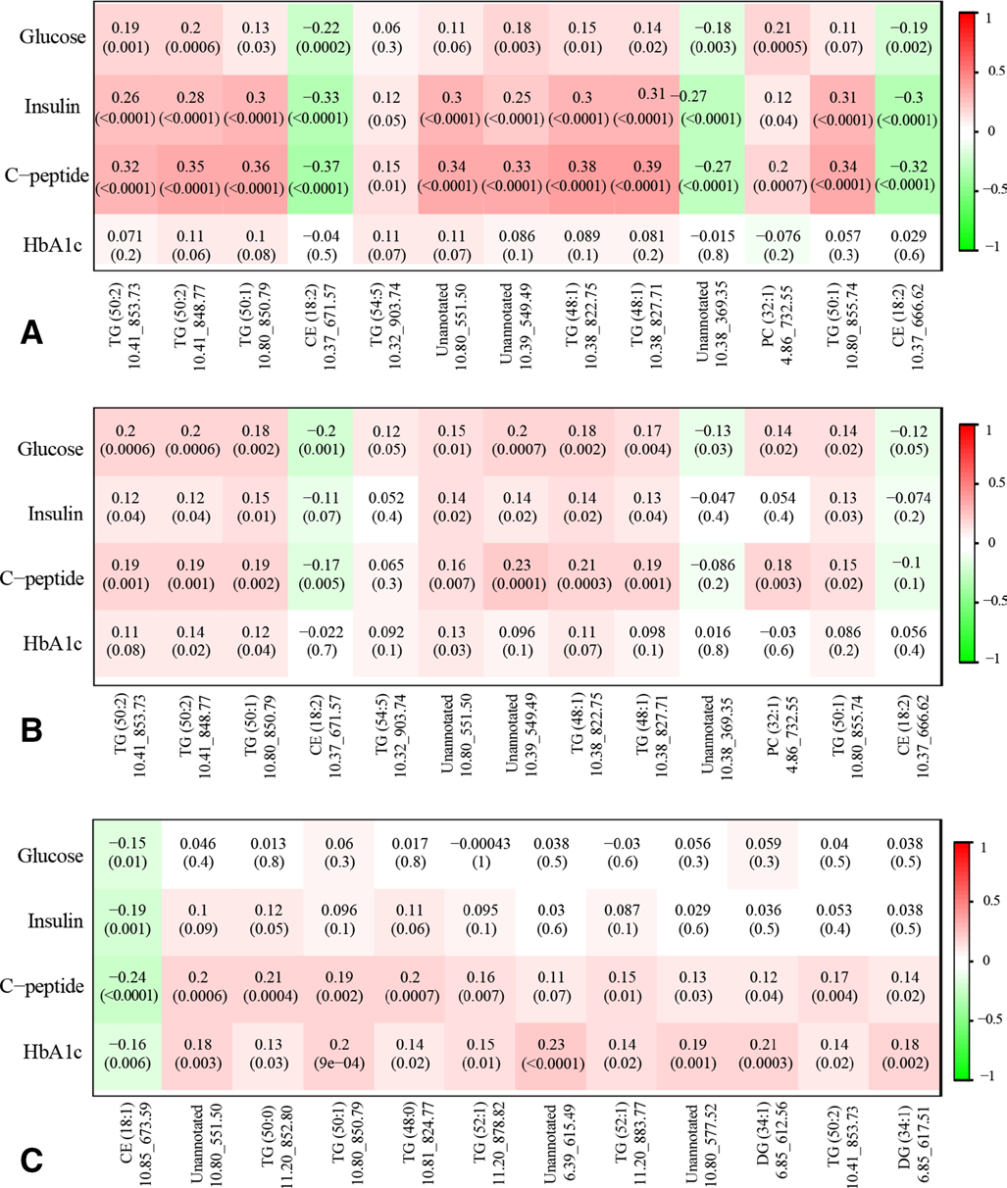
Pearson’s partial correlation coefficients (p values) between GDM-related selected lipid metabolites with maternal glycemic markers, adjusted for maternal age, enrollment BMI, family history of diabetes, alcohol use before pregnancy, race/ethnicity and gestational age at blood collection. (A) Cross-sectional associations of selected lipid metabolites with non-fasting glycemic markers at visit 0; (B) Prospective associations of selected lipid metabolites at visit 0 with fasting glycemic markers at visit 1; (C) Cross-sectional associations of selected lipid metabolites and fasting glycemic markers at visit 1. BMI, body mass index; CE, cholesteryl ester; DG, diglyceride; GDM, gestational diabetes; HbA1c, glycated hemoglobin; TG, triglyceride.

GDM-related individual lipid metabolites also showed consistent correlations with maternal glycemic biomarkers (figure 3). For example, plasma concentration of TG (50:1; 10.80_850.79) was significantly and positively correlated with plasma glucose (r=0.19, p=0.001), insulin (r=0.26, p<0.0001) and C-peptide (r=0.32, p<0.0001) in cross-sectional analyses at visit 0, whereas CE (18:2; 10.37_671.57) was significantly and negatively correlated with glucose (r=−0.22, p<0.0002), insulin (r=−0.33, p<0.0001) and C-peptide (r=−0.37, p<0.0001). A similar but weaker correlation pattern was observed in prospective analyses of metabolites measured at visit 0 with fasting plasma glucose, insulin, and C-peptide measured at visit 1. In cross-sectional analyses at visit 1, we observed a consistent correlation pattern particularly for fasting plasma C-peptide and HbA1c.

### Temporal trend of GDM-related individual lipid metabolites across pregnancy

Leveraging on longitudinally collected lipidomics data, we further examined plasma concentrations of selected GDM-related metabolites between cases and controls across pregnancy (online supplemental figure S3). Overall, the difference in mean metabolite concentration by case-control status, if any, was mostly observed before the diagnosis of GDM (24–28 weeks), which progressively attenuated in late pregnancy. For example, the mean plasma concentration of TG (50:2), TG (50:0), TG (50:1) and TG (48:0) were significantly higher among GDM cases compared with non-GDM controls at visit 0 and visit 1, whereas the opposite pattern was observed for CE (18:1) and CE (18:2).

10.1136/bmjdrc-2020-001551.supp5 Supplementary data

### Sensitivity analyses

Sensitivity analysis additionally adjusting the models for maternal smoking prior to pregnancy did not change the associations between lipid networks and GDM risk at both study visits. Additional sensitivity analysis stratified by GDM treatment status suggested that the associations between lipid networks and GDM risk may be influenced by disease severity status as early as 10-14 weeks. Overall, we observed consistent associations for lipid networks with GDM risk among women who developed GDM but did not require medical intervention (insulin or other medication) at both study visits. For example, the ‘turquoise’ and ‘blue’ networks showed consistent negative associations with GDM at both study visits, whereas the ‘yellow’ and ‘brown’ network showed positive associations with GDM risk at visit 0 among women who developed GDM but did not require medical intervention (online supplemental table S2). No association was observed among women who developed GDM and required intervention using insulin or other medication.

10.1136/bmjdrc-2020-001551.supp6 Supplementary data

## Discussion

In this prospective study of 420 untargeted lipid metabolites in the plasma of multirace/ethnic US women in early pregnancy to mid-pregnancy, we observed that several plasma glycerolipids, some phospholipids and sterol lipids and SMs in early pregnancy both individually and interactively in distinct lipid networks were associated with subsequent risk of developing GDM. Overall, these GDM-related lipid metabolites and lipid networks also showed moderate to weak but consistent correlations with maternal glycemic biomarkers in early pregnancy to mid-pregnancy. Our results also suggested that acyl carbon chain length, double bond characteristics of the lipid metabolites may play an important role in maternal cardiometabolic risk.

Previous studies on maternal plasma lipidomics and GDM are sparse. We are aware of only two studies that have prospectively examined the association among predominantly Caucasian white^13^ or Han Chinese populations.^12^ Unlike our study, those two studies have implemented guidelines proposed by the International Association of Diabetes and Pregnancy Study Groups (IADPSG), which adopted a more stringent criterion for GDM diagnosis^28^ and in most populations lead to a significant increase in the number of women labeled as GDM.^29^ Hence, IADPSG criteria may potentially lead to classifying some women as having GDM who would be considered as having normal glucose tolerance by other diagnostic criteria (ie, Carpenter and Coustan criteria), and as such might reduce the variability in metabolite concentrations between GDM cases and non-GDM controls. Nonetheless, our findings are in consistent with previous prospective studies in other populations. In the UK study among predominantly white Caucasians, higher concentrations of glycerolipids, particularly TG (48:1), TG (51:1) and low-saturated phospholipid, PC (32:1) in the plasma in early second trimester have been associated with higher GDM risk.^13^ These findings are also in consistent with reports in non-pregnant population where glycerolipids, such as DGs and short, saturated/low unsaturated TGs have been positively associated with insulin resistance, T2D and cardiovascular diseases.^8 27^ Cellular accumulation of DG, which is an intermediate of TG metabolism,^30^ has been associated with obesity and insulin resistance,^31 32^ mediated via protein kinase C activation.^33^ TGs and elevated free fatty acids have also been implicated in insulin resistance.^30^ Free fatty acids can generate oxidative stress,^34^ activating protein kinase C and thereby, contribute to insulin resistance.^35^ Our observed positive associations of several glycerolipids with GDM and maternal fasting and non-fasting glycemic biomarkers thus, reinforce existing literature on the etiological role of glycerolipids in the development of GDM.

In contrast, phospholipids rich with unsaturated fatty acids have been negatively associated with GDM.^12 36 37^ In Han Chinese population, women who subsequently developed GDM had a lower concentration of several polyunsaturated PCs and PEs in the first trimester plasma.^12^ PCs rich with polyunsaturated fatty acids have also been negatively correlated with postload glucose, HbA1c, homeostatic model assessment of insulin resistance (HOMA-IR) and type 2 diabetes in non-pregnant population.^38^ In this study, we observed marked heterogeneity in associations between PCs and GDM based on the type of acyl carbon double bond to the glycerol backbone, where higher plasma concentrations of PCs with ester-linkage or ether-linkage were associated with lower risk of GDM. This pattern have also been reported in non-pregnant populations, where higher plasmenyl-phospholipids consisting of *O*-alk-1′-enyl linkage (also known as plasmalogen) have been associated with higher insulin sensitivity, lower insulin secretion and higher risk of type 2 diabetes.^39 40^ Plasmalogen is an essential constituent of animal lipid membrane^41^ and act as an antioxidant to prevent lipoprotein oxidation.^39^ They also have anti-apoptotic and anti-inflammatory property,^42^ which may play a role in minimizing the risk for GDM.

SMs are structurally similar to PCs but contain ceramides instead of diacylglycerols.^43^ SMs are involved in plasma membrane signal transduction, cholesterol efflux and intracellular lipid and protein trafficking.^44^ Similar to our study, prior studies have also reported negative correlations between SMs and maternal fasting and 2 hour postload glucose, fasting insulin and HOMA-IR among women with a history of GDM.^10^ These findings are reinforced by evidence in non-pregnant population where lower concentrations of SMs have been associated with higher HOMA-IR, homeostatic model assessment of β-cell function (HOMA-beta), fasting insulin and higher risk of type 2 diabetes^40 45^ and transitioning to type 2 diabetes from GDM.^46^ Animal studies have demonstrated that downregulation of sphingolipid metabolism in mouse islets and pancreatic beta-cell like cell lines have been associated with impaired glucose-stimulated insulin secretion without considerably impacting whole-body insulin sensitivity or glucose homeostasis.^46^

Similar to phospholipids and sphingolipids, our study demonstrated that higher concentrations of CEs in early pregnancy to mid-pregnancy were associated with lower risk of GDM. CEs are long-chain fatty acids linked to hydroxyl group of cholesterol, where plasma CEs tend to contain a relatively high proportion of polyunsaturated fatty acid.^47^ There are sparse data on CEs with GDM risk in pregnant population, although higher concentrations of CEs have been associated with lower risk of type 2 diabetes and cardiovascular diseases in non-pregnant populations,^8 26^ suggesting an overall beneficial effect of CEs on cardiometabolic health.

Our results suggest that GDM severity status may influence the associations between lipidomics and GDM risk as early as in early pregnancy. Women who were identified having severe disease requiring intervention using insulin or other medications at the time of diagnosis might have developed subclinical disease or pre-existing metabolic abnormality in early pregnancy, which could dysregulate lipid metabolites prior to the diagnosis of GDM. Furthermore, the therapeutic effect of GDM intervention could also influence lipidomics-GDM associations. The diminished difference in plasma concentrations of GDM-related metabolites between cases and controls could presumably be attributable to GDM treatment effect, which further suggests potential etiological role of lipid metabolites in GDM. Given the notable changes in concentrations of GDM-related lipid metabolites across gestation as observed herein, and potential influence of the therapeutic effect of GDM intervention after the diagnosis, it is important to investigate the time-specific associations prior to GDM screening.

Our study has several unique strengths. We analyzed a comprehensive spectrum of lipid metabolites in relation to GDM risk in a multiracial/ethnic population as opposed to previous prospective studies^12 13^ that were conducted on a single race/ethnic group. Our prospective and longitudinal collection of lipidomics data allowed us to examine the temporal precedence in lipidomics and GDM associations. In particular, the case-control differences in most GDM-related lipid metabolites did not persist after the average gestational age at GDM diagnosis (ie, approximately week 27), which highlights the importance of temporal precedence in investigating the etiological roles of lipid metabolites. Furthermore, we had the unique ability to profile the longitudinal trends of lipid metabolites across pregnancy, which demonstrated differential temporal variations of the metabolites between women with and without GDM. We collected comprehensive data on maternal demographic and medical history to account for major GDM risk factors in our analysis. The overall consistent associations at both study visits suggest that our observed findings are internally replicable. In addition, we implemented a novel network analysis approach to investigate how metabolites within intertwined networks impact GDM risk via comprehensive interactions.^21^ Lastly, we analyzed a comprehensive panel of glycemic biomarkers with lipid metabolites to shed lights on the role of lipid metabolites into GDM etiology.

Some potential limitations of our study merit discussion. Plasma lipidome was measured using untargeted approach, which merits validation in another study and possibly using targeted approach. In this study, the plasma samples at 10–14 weeks were obtained in non-fasting state without controlling for mealtime and meal content. Hence, additional variability in lipid measurements might have introduced that was not related to variation in OGTT results at 28 weeks of gestation, thus reducing study power. However, this additional variability was likely small compared with the overall variability as within-subject variability in lipidomic profiles attributed to mealtime (≈7%) were much smaller than the within-subject variability not attributed to mealtime (≈31%) or the between-subject variability (≈62%),^48^ albeit in a population less diverse in race/ethnicity than ours. Although this is the first study of its kind conducted in a race/ethnically diverse pregnant population, due to small sample size, we did not conduct stratified analysis by race/ethnicity to examine the effect of ethnicity on the lipidome. Finally, the generalization of our study findings to the overall US pregnant population remains to be established, as the NICHD Fetal Growth Study enrolled pregnant women with low-risk prenatal profiles without major pre-existing chronic conditions. However, inclusion of overall healthy women may minimize reverse causality and residual confounding due to pre-existing complications and unhealthy lifestyle factors.

In this prospective study among pregnant women of multirace/ethnic groups with longitudinal measurement of plasma lipidome across pregnancy, we report that early pregnancy plasma concentrations of a number of glycerolipids and some phospholipid and sterol lipids and sphingomyelins both individually and interactively in distinct networks were prospectively associated with subsequent GDM risk. These GDM-related lipid metabolites and lipid networks showed consistent correlation pattern with maternal fasting and non-fasting glycemic biomarkers. If confirmed, these finding shed light on potential role of lipid metabolites as etiological biomarkers of GDM.
