# Supplementary material for: Plasma lipidomics profile in pregnancy and gestational diabetes risk: a prospective study in a multiracial/ethnic cohort
Source: BMJ Open Diabetes Res Care. 2021 Mar 5;9(1):e001551. doi: 10.1136/bmjdrc-2020-001551 (PMC7939004; doi:10.1136/bmjdrc-2020-001551)
Supplement: Supplementary data [file bmjdrc-2020-001551supp001.pdf]

Supplementary Data

Plasma lipidomics profile in pregnancy and gestational diabetes risk- a prospective study in a multi-racial/ethnic cohort

Mohammad L. Rahman[1,2*], Yen-Chen A. Feng[3,4*], Oliver Fiehn[5], Paul S. Albert[6], Michael Y Tsai[7], Yeyi Zhu[8], Xiaobin Wang[9,10], Fasil Tekola-Ayele[1], Liming Liang[11], Cuilin Zhang[1]

Corresponding authors:
Cuilin Zhang, MD, PhD, MPH
Division of Intramural Population Health Research, *Eunice Kennedy Shriver* National Institute of Child Health and Human Development, National Institutes of Health
6710B Rockledge Drive, MSC 7004, Bethesda, MD 20817.
Phone: (301) 435-6917
E-mail: zhangcu@mail.nih.gov
ORCID ID: 0000-0002-8014-2708

Liming Liang, PhD
Departments of Epidemiology and Biostatistics
Harvard T.H. Chan School of Public Health
655 Huntington Ave, Building 2-207, Boston, MA 02115
Phone: 6174325896
E-mail: lliang@hsph.harvard.edu

General Business

Supplementary table S1. Individual lipid metabolites and their associations with gestational diabetes (GDM) risk by study visit.

Supplementary table S2. Associations of lipid networks with GDM risk stratified by disease severity at visit 1 (15-26 weeks).

Supplementary figure S1. Flow chart of the nested case–control study, within the NICHD Fetal Growth Studies–Singleton Cohort. NICHD = National Institute of Child Health and Human Development.

Supplementary figure S2. Scatterplots showing prospective associations between individual lipid metabolites and subsequent GDM risk by number of acyl carbons (x-axis) and double bonds (y-axis) by lipid classes at visit 0 (**A**) and visit 1 (**B**). Each circle represents individual lipid metabolite-GDM relations, where the color gradient of the circle represents the direction of association (red= positive associations; blue= negative associations), and the size of the circle represents -log10(p-value). Associations were adjusted for maternal age, enrollment BMI, family history of diabetes, alcohol use before pregnancy, race/ethnicity, and gestational age at blood collection.

Supplementary figure S3. Longitudinal profiles (mean concentrations ± standard errors) of selected metabolites between GDM cases and non-GDM controls across pregnancy. Annotated and unannotated metabolites that showed most significant and consistent associations with GDM

General Business

at both study visits were selected for visualization. *P <0.05; ** P <0.01; *** P <0.001 are test statistics for between-group comparisons at each gestational interval

General Business
