# Supplementary material for: Plasma lipidomics profile in pregnancy and gestational diabetes risk: a prospective study in a multiracial/ethnic cohort
Source: BMJ Open Diabetes Res Care. 2021 Mar 5;9(1):e001551. doi: 10.1136/bmjdrc-2020-001551 (PMC7939004; doi:10.1136/bmjdrc-2020-001551)
Supplement: Supplementary data [file bmjdrc-2020-001551supp002.pdf]

Supplementary figure S1. Flow chart of the nested case-control study, within the NICHD Fetal Growth Studies-Singleton Cohort

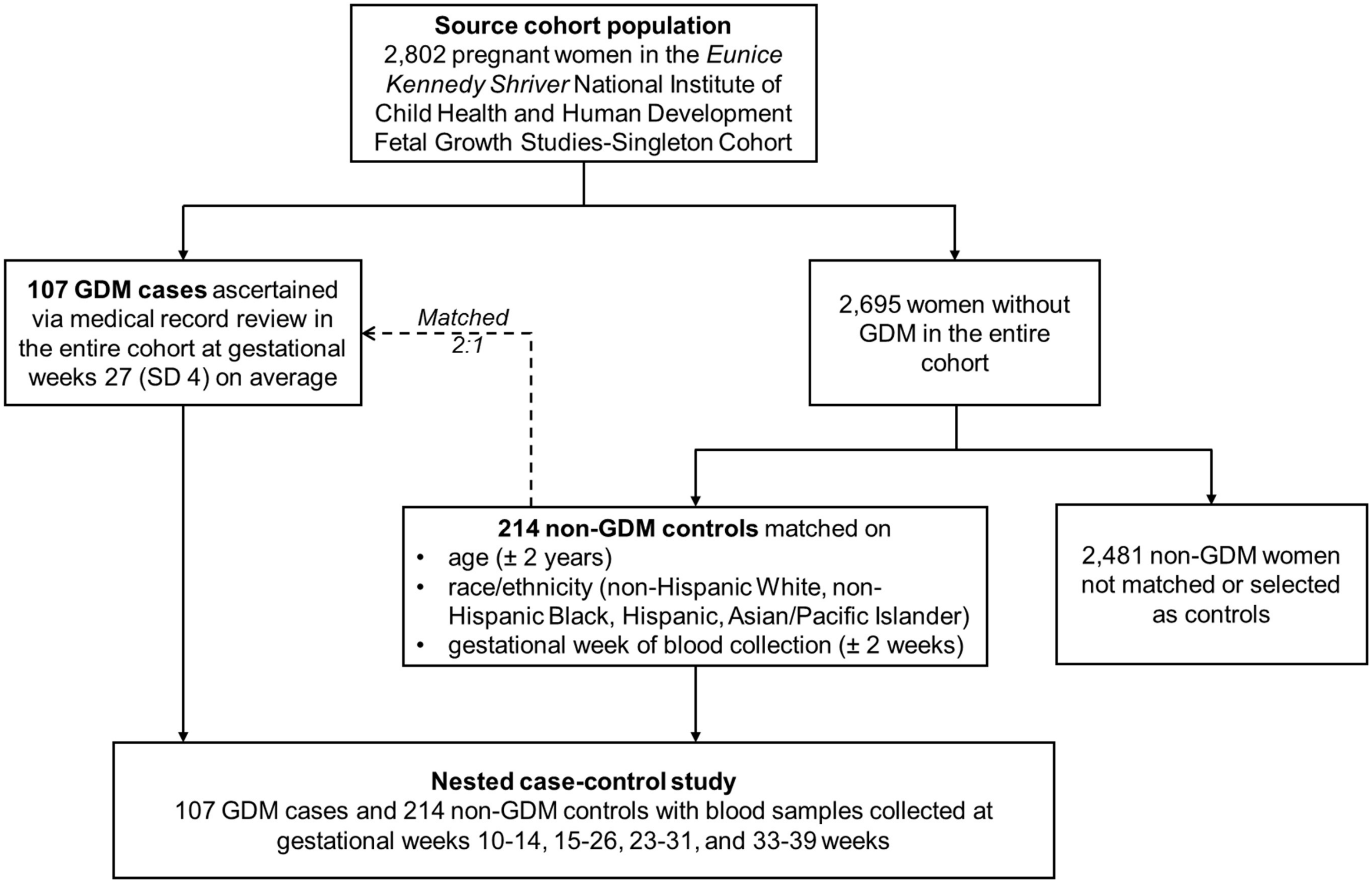

Rahman ML, *et al. BMJ Open Diab Res Care* 2021; 9:e001551. doi: 10.1136/bmjdrc-2020-001551
