# Supplementary material for: Plasma lipidomics profile in pregnancy and gestational diabetes risk: a prospective study in a multiracial/ethnic cohort
Source: BMJ Open Diabetes Res Care. 2021 Mar 5;9(1):e001551. doi: 10.1136/bmjdrc-2020-001551 (PMC7939004; doi:10.1136/bmjdrc-2020-001551)
Supplement: Supplementary data [file bmjdrc-2020-001551supp003.pdf]

Table S1. Individual lipid metabolites and their associations with gestational diabetes (GDM) risk by study visit.

			Visit 0: 10-14 weeks			Visit 1: 15-26 weeks		
**Lipid Class**	**Annotation**	**RT_m/z**	**estimate**	***P*-value**	**FDR**	**estimate**	***P*-value**	**FDR**
**Acylcarnitines**								
	Acylcarnitine C16:0	1.58_400.34	0.07	5.5E-01	7.3E-01	0.19	1.1E-01	2.3E-01
	Acylcarnitine C10:0	0.58_316.25	-0.08	4.9E-01	6.8E-01	-0.09	4.1E-01	5.5E-01
	Acylcarnitine C12:0	0.73_344.28	-0.08	4.7E-01	6.6E-01	-0.09	4.6E-01	5.9E-01
	Acylcarnitine C18:1	1.73_426.36	-0.10	4.1E-01	6.1E-01	0.05	6.5E-01	7.6E-01
	Acylcarnitine C18:0	2.33_428.37	-0.11	3.7E-01	5.6E-01	-0.03	8.1E-01	8.6E-01
	Acylcarnitine C18:2	1.33_424.34	-0.16	2.1E-01	3.7E-01	0	9.9E-01	9.9E-01
**Glycerolipids (GL)**								
**Diglycerides (DG)**								
	DG (34:1)	6.85_612.56	0.44	1.6E-04	**4.8E-03**	0.45	7.3E-05	**3.1E-03**
	DG (34:1)	6.85_617.51	0.42	3.1E-04	**5.9E-03**	0.43	1.2E-04	**3.7E-03**
	DG (36:3)	6.40_641.51	0.18	1.3E-01	2.9E-01	0.34	4.2E-03	**2.6E-02**
	DG (36:3)	6.40_636.56	0.18	1.4E-01	3.1E-01	0.32	5.0E-03	**2.8E-02**
	DG (36:2)	6.93_643.53	0.29	1.7E-02	8.3E-02	0.32	6.6E-03	**3.3E-02**
	DG (36:2)	6.93_638.57	0.34	4.0E-03	**3.4E-02**	0.3	9.0E-03	**4.0E-02**
	DG (38:5)	6.25_665.51	0.39	7.5E-04	**1.2E-02**	0.29	1.4E-02	5.8E-02
	DG (38:6)	5.71_663.50	0.24	3.8E-02	1.3E-01	0.28	2.3E-02	8.4E-02
	DG (38:5)	6.25_660.56	0.38	1.0E-03	**1.4E-02**	0.23	4.8E-02	1.3E-01
	DG (38:6)	5.71_658.54	0.34	3.3E-03	**3.0E-02**	0.24	4.9E-02	1.3E-01
**Triglycerides (TG)**								
	TG (50:1)	10.80_850.79	0.54	2.2E-06	**3.0E-04**	0.51	6.1E-06	**6.4E-04**
	TG (50:0)	11.20_852.80	0.30	8.5E-03	5.6E-02	0.53	5.8E-06	**6.4E-04**
	TG (48:0)	10.81_824.77	0.41	4.3E-04	**7.2E-03**	0.52	9.5E-06	**7.9E-04**
	TG (52:1)	11.20_878.82	0.43	2.2E-04	**5.4E-03**	0.5	1.5E-05	**1.1E-03**
	TG (52:1)	11.20_883.77	0.42	2.9E-04	**5.9E-03**	0.48	3.1E-05	**1.6E-03**
	TG (50:2)	10.41_853.73	0.55	8.5E-07	**3.0E-04**	0.43	1.1E-04	**3.7E-03**
	TG (50:2)	10.41_848.77	0.54	1.8E-06	**3.0E-04**	0.43	1.3E-04	**3.7E-03**
	TG (58:6)	10.65_952.83	0.41	7.5E-04	**1.2E-02**	0.44	1.3E-04	**3.7E-03**
	TG (54:1)	11.56_906.85	0.35	3.8E-03	**3.3E-02**	0.45	1.6E-04	**4.2E-03**
	TG (54:5)	10.32_903.74	0.50	1.6E-05	**1.1E-03**	0.44	1.8E-04	**4.3E-03**
	TG (50:3)	10.01_846.76	0.42	2.4E-04	**5.4E-03**	0.41	2.2E-04	**4.6E-03**
	TG (52:0)	11.56_880.83	0.28	1.9E-02	8.6E-02	0.44	2.2E-04	**4.6E-03**
	TG (52:4)	10.09_872.77	0.18	1.2E-01	2.8E-01	0.43	2.3E-04	**4.6E-03**
	TG (58:6)	10.65_957.79	0.44	3.0E-04	**5.9E-03**	0.43	2.8E-04	**4.7E-03**
	TG (54:2)	11.20_904.83	0.36	3.0E-03	**2.8E-02**	0.43	3.1E-04	**4.7E-03**
	TG (49:0)	10.96_838.79	0.28	1.5E-02	7.6E-02	0.42	3.1E-04	**4.7E-03**
	TG (50:0)	11.20_857.76	0.24	3.5E-02	1.2E-01	0.43	3.0E-04	**4.7E-03**
	TG (52:5)	9.71_870.76	0.24	4.0E-02	1.3E-01	0.43	2.7E-04	**4.7E-03**
	TG (48:1)	10.38_822.75	0.48	3.1E-05	**1.6E-03**	0.42	3.4E-04	**4.9E-03**
	TG (56:4)	10.88_933.79	0.33	4.9E-03	**3.9E-02**	0.4	3.8E-04	**4.9E-03**
	TG (52:2)	10.88_876.80	0.29	1.6E-02	7.8E-02	0.41	3.5E-04	**4.9E-03**
	TG (56:3)	11.20_930.85	0.28	2.6E-02	1.0E-01	0.4	4.4E-04	**5.6E-03**
	TG (50:1)	10.80_855.74	0.47	8.8E-05	**2.9E-03**	0.41	4.8E-04	**5.9E-03**
	TG (52:3)	10.46_874.79	0.24	4.9E-02	1.5E-01	0.38	5.7E-04	**6.8E-03**
	TG (48:0)	10.81_829.73	0.32	6.7E-03	**4.8E-02**	0.41	6.3E-04	**7.1E-03**
	TG (54:1)	11.56_911.80	0.32	7.1E-03	5.1E-02	0.4	6.2E-04	**7.1E-03**
	TG (49:0)	10.96_843.74	0.25	3.1E-02	1.1E-01	0.4	6.6E-04	**7.1E-03**
	TG (46:0)	10.36_796.74	0.37	1.3E-03	**1.7E-02**	0.41	6.9E-04	**7.3E-03**
	TG (53:1)	11.35_897.79	0.30	1.0E-02	6.1E-02	0.39	7.4E-04	**7.3E-03**
	TG (52:5)	9.71_875.71	0.26	2.8E-02	1.0E-01	0.4	7.1E-04	**7.3E-03**
	TG (54:6)	9.95_901.73	0.27	2.4E-02	9.8E-02	0.38	8.3E-04	**7.7E-03**
	TG (56:4)	10.88_928.83	0.15	2.2E-01	4.1E-01	0.37	8.4E-04	**7.7E-03**
	TG (54:6)	9.95_896.77	0.37	9.9E-04	**1.4E-02**	0.39	9.2E-04	**8.3E-03**
	TG (51:1)	10.93_869.76	0.37	2.0E-03	**2.2E-02**	0.36	1.5E-03	**1.2E-02**
	TG (51:1)	10.93_864.80	0.36	2.1E-03	**2.2E-02**	0.36	1.5E-03	**1.2E-02**

	TG (56:1)	11.85_934.88	0.19	1.2E-01	2.7E-01	0.36	1.4E-03	**1.2E-02**
	TG (56:2)	11.56_937.82	0.24	4.3E-02	1.4E-01	0.37	1.7E-03	**1.3E-02**
	TG (56:2)	11.56_932.86	0.21	8.4E-02	2.1E-01	0.36	2.0E-03	**1.5E-02**
	TG (48:1)	10.38_827.71	0.48	3.5E-05	**1.7E-03**	0.36	2.3E-03	**1.7E-02**
	TG (50:3)	10.01_851.71	0.37	9.2E-04	**1.3E-02**	0.33	2.4E-03	**1.7E-02**
	TG (48:2)	9.98_820.74	0.43	2.4E-04	**5.4E-03**	0.34	3.5E-03	**2.3E-02**
	TG (56:6)	10.32_929.76	0.25	2.7E-02	1.0E-01	0.34	3.9E-03	**2.5E-02**
	TG (52:0)	11.56_885.79	0.11	3.4E-01	5.5E-01	0.34	4.6E-03	**2.7E-02**
	TG (58:4)	11.22_956.86	0.27	2.6E-02	1.0E-01	0.33	5.5E-03	**3.0E-02**
	TG (51:3)	10.23_860.77	0.28	1.4E-02	7.4E-02	0.31	6.2E-03	**3.2E-02**
	TG (56:6)	10.32_924.80	0.25	2.7E-02	1.0E-01	0.31	6.1E-03	**3.2E-02**
	TG (50:4)	9.61_844.74	0.24	3.9E-02	1.3E-01	0.32	5.9E-03	**3.2E-02**
	TG (56:1)	11.85_939.84	0.25	3.0E-02	1.1E-01	0.31	6.8E-03	**3.3E-02**
	TG (58:3)	11.58_958.88	0.21	7.1E-02	1.9E-01	0.31	6.7E-03	**3.3E-02**
	TG (56:8)	9.76_920.77	0.16	1.9E-01	3.7E-01	0.3	8.6E-03	**3.9E-02**
	TG (51:2)	10.61_862.79	0.23	5.3E-02	1.5E-01	0.3	8.9E-03	**4.0E-02**
	TG (49:1)	10.59_836.77	0.32	5.4E-03	**4.1E-02**	0.28	1.1E-02	**4.7E-02**
	TG (58:1)	11.99_962.91	0.22	5.7E-02	1.6E-01	0.29	1.2E-02	5.0E-02
	TG (58:2)	11.85_965.85	0.21	7.5E-02	1.9E-01	0.28	1.2E-02	5.2E-02
	TG (53:1)	11.35_892.83	0.33	4.9E-03	**3.9E-02**	0.31	1.2E-02	5.3E-02
	TG (50:4)	9.61_849.69	0.23	5.1E-02	1.5E-01	0.29	1.3E-02	5.3E-02
	TG (54:3)	10.84_902.82	0.05	7.0E-01	8.2E-01	0.29	1.3E-02	5.5E-02
	TG (58:4)	11.22_961.82	0.30	1.1E-02	6.5E-02	0.29	1.4E-02	5.7E-02
	TG (49:2)	10.18_834.76	0.35	2.1E-03	**2.2E-02**	0.27	1.5E-02	5.8E-02
	TG (58:2)	11.85_960.90	0.14	2.4E-01	4.3E-01	0.26	1.8E-02	6.8E-02
	TG (50:5)	9.21_847.68	0.29	1.5E-02	7.6E-02	0.27	2.1E-02	7.8E-02
	TG (50:5)	9.21_842.72	0.27	2.1E-02	9.4E-02	0.27	2.1E-02	7.8E-02
	TG (48:2)	9.98_825.69	0.36	2.3E-03	**2.4E-02**	0.27	2.3E-02	8.3E-02
	TG (53:4)	10.31_886.79	0.13	2.8E-01	4.7E-01	0.27	2.3E-02	8.3E-02
	TG (49:3)	9.79_832.74	0.29	9.9E-03	6.1E-02	0.25	2.6E-02	8.8E-02
	TG (49:1)	10.59_841.73	0.29	1.2E-02	6.5E-02	0.25	2.6E-02	8.8E-02
	TG (49:2)	10.18_839.71	0.29	1.3E-02	6.9E-02	0.24	2.6E-02	8.8E-02
	TG (56:8)	9.76_925.73	0.18	1.4E-01	3.0E-01	0.27	2.6E-02	8.8E-02
	TG (54:3)	10.84_907.77	0.13	2.7E-01	4.7E-01	0.25	2.7E-02	9.0E-02
	TG (58:1)	11.99_967.87	0.27	2.2E-02	9.7E-02	0.24	2.8E-02	9.1E-02
	TG (60:2)	11.99_988.93	0.12	3.0E-01	5.0E-01	0.25	2.8E-02	9.1E-02
	TG (60:2)	11.99_993.88	0.17	1.4E-01	3.0E-01	0.24	2.9E-02	9.2E-02
	TG (52:6)	9.35_873.69	0.19	1.2E-01	2.7E-01	0.25	3.3E-02	9.9E-02
	TG (46:1)	9.93_794.72	0.36	2.5E-03	**2.5E-02**	0.25	3.5E-02	1.0E-01
	TG (54:8)	9.05_892.74	0.29	1.0E-02	6.1E-02	0.25	3.6E-02	1.0E-01
	TG (52:4)	10.09_877.73	-0.02	8.8E-01	9.5E-01	0.23	3.5E-02	1.0E-01
	TG (58:3)	11.58_963.84	0.15	2.0E-01	3.8E-01	0.24	3.8E-02	1.1E-01
	TG (53:3)	10.64_888.80	0.14	2.4E-01	4.4E-01	0.24	4.1E-02	1.1E-01
	TG (53:2)	11.00_890.82	0.19	1.2E-01	2.6E-01	0.24	4.2E-02	1.2E-01
	TG (56:7)	9.96_922.79	0.18	1.2E-01	2.7E-01	0.23	4.7E-02	1.3E-01
	TG (56:5)	10.77_931.77	0.18	1.3E-01	2.9E-01	0.23	5.3E-02	1.4E-01
	TG (58:9)	9.79_946.79	0.17	1.6E-01	3.3E-01	0.22	5.4E-02	1.4E-01
	TG (51:4)	9.85_858.75	0.15	1.9E-01	3.7E-01	0.23	5.3E-02	1.4E-01
	TG (54:6)	9.70_896.77	0.15	2.2E-01	4.2E-01	0.23	5.4E-02	1.4E-01
	TG (54:4)	10.48_900.80	0.04	7.6E-01	8.6E-01	0.23	5.4E-02	1.4E-01
	TG (58:10)	9.40_944.77	0.24	4.0E-02	1.3E-01	0.22	6.3E-02	1.5E-01
	TG (51:3)	10.23_865.73	0.17	1.4E-01	3.0E-01	0.22	5.9E-02	1.5E-01
	TG (58:8)	10.15_948.80	0.14	2.5E-01	4.4E-01	0.21	6.2E-02	1.5E-01
	TG (56:3)	11.20_935.80	0.08	5.0E-01	6.9E-01	0.21	6.3E-02	1.5E-01
	TG (53:5)	9.86_884.77	0.08	4.9E-01	6.8E-01	0.21	7.4E-02	1.7E-01
	TG (48:3)	9.53_818.72	0.34	3.7E-03	**3.3E-02**	0.2	8.6E-02	1.9E-01
	TG (46:3)	9.00_790.69	0.22	6.1E-02	1.7E-01	0.21	8.4E-02	1.9E-01
	TG (46:1)	9.93_799.68	0.31	1.0E-02	6.1E-02	0.2	9.2E-02	2.0E-01
	TG (46:2)	9.50_792.71	0.30	1.3E-02	6.9E-02	0.2	9.3E-02	2.0E-01

	TG (44:1)	9.43_766.69	0.27	2.2E-02	9.7E-02	0.2	1.0E-01	2.2E-01
	TG (51:2)	10.61_867.74	0.30	8.5E-03	5.6E-02	0.18	1.2E-01	2.3E-01
	TG (49:3)	9.80_837.69	0.25	2.8E-02	1.0E-01	0.18	1.1E-01	2.3E-01
	TG (46:3)	9.00_795.65	0.21	8.3E-02	2.1E-01	0.19	1.1E-01	2.3E-01
	TG (53:2)	11.00_895.77	0.22	6.8E-02	1.8E-01	0.18	1.2E-01	2.4E-01
	TG (54:4)	10.48_905.76	0.03	8.3E-01	9.2E-01	0.18	1.2E-01	2.4E-01
	TG (14:0/14:0/14:0)	9.43_740.68	0.24	4.9E-02	1.5E-01	0.19	1.3E-01	2.5E-01
	TG (46:2)	9.50_797.66	0.28	2.1E-02	9.4E-02	0.18	1.4E-01	2.6E-01
	TG (58:10)	9.40_949.73	0.13	2.8E-01	4.7E-01	0.18	1.4E-01	2.6E-01
	TG (60:11)	9.65_970.79	0.22	6.7E-02	1.8E-01	0.17	1.5E-01	2.8E-01
	TG (58:8)	10.15_953.76	-0.05	6.7E-01	8.0E-01	0.16	1.7E-01	3.1E-01
	TG (54:5)	10.10_898.79	0.08	5.4E-01	7.2E-01	0.16	1.8E-01	3.2E-01
	TG (51:4)	9.85_863.71	0.01	9.2E-01	9.7E-01	0.16	1.9E-01	3.3E-01
	TG (48:3)	9.53_823.68	0.33	5.7E-03	**4.2E-02**	0.15	2.0E-01	3.4E-01
	TG (53:5)	9.86_889.73	-0.02	8.4E-01	9.2E-01	0.15	2.0E-01	3.5E-01
	TG (44:1)	9.43_771.65	0.24	4.5E-02	1.4E-01	0.15	2.2E-01	3.6E-01
	TG (56:7)	9.96_927.74	-0.08	5.0E-01	6.9E-01	0.13	2.5E-01	4.0E-01
	TG (58:9)	9.79_951.74	0.12	3.1E-01	5.1E-01	0.13	2.7E-01	4.2E-01
	TG (54:5)	10.10_903.74	0.05	6.6E-01	8.0E-01	0.13	2.6E-01	4.2E-01
	TG (54:6)	9.70_901.73	0.03	8.3E-01	9.2E-01	0.13	2.7E-01	4.2E-01
	TG (52:6)	9.35_868.74	0.22	6.1E-02	1.7E-01	0.13	2.8E-01	4.4E-01
	TG (53:4)	10.31_891.74	-0.09	4.4E-01	6.4E-01	0.11	3.6E-01	5.0E-01
	TG (54:8)	9.05_897.69	0.05	6.6E-01	7.9E-01	0.11	3.6E-01	5.1E-01
	TG (53:3)	10.64_893.76	-0.01	9.6E-01	9.7E-01	0.1	3.9E-01	5.3E-01
	TG (54:2)	11.20_909.79	0.17	1.6E-01	3.4E-01	0.09	4.5E-01	5.8E-01
	TG (54:5)	10.32_898.79	0.26	3.2E-02	1.1E-01	0.04	7.5E-01	8.2E-01
	TG (14:0/14:0/14:0)	9.43_745.63	0.01	9.5E-01	9.7E-01	0.03	8.1E-01	8.6E-01
	TG (52:2)	10.88_881.76	0.11	3.6E-01	5.6E-01	-0.02	8.8E-01	9.2E-01
	TG (52:3)	10.46_879.74	0.06	5.5E-01	7.3E-01	0.01	9.3E-01	9.5E-01
	TG (56:5)	10.77_926.82	-0.05	7.0E-01	8.2E-01	-0.01	9.6E-01	9.8E-01
**Glycerophospholipids (GP)**								
**Phosphatidylcholines (PC)**								
	PC (38:3)	5.79_812.61	0.42	2.0E-04	**5.4E-03**	0.36	3.1E-03	**2.0E-02**
	PC (p-34:1) or PC (o-34:2) B	5.79_744.58	-0.16	1.1E-01	2.6E-01	-0.32	4.4E-03	**2.6E-02**
	PC (37:6)	4.50_792.55	-0.12	2.8E-01	4.7E-01	-0.3	4.4E-03	**2.6E-02**
	PC (39:6)	4.99_820.59	-0.23	3.3E-02	1.2E-01	-0.31	4.6E-03	**2.7E-02**
	PC (p-34:2) or PC (o-34:3)	5.27_742.58	-0.12	2.7E-01	4.7E-01	-0.29	8.3E-03	**3.9E-02**
	PC (40:7)	4.82_832.59	-0.21	5.2E-02	1.5E-01	-0.25	1.4E-02	5.8E-02
	PC (37:2)	5.92_800.62	-0.20	7.8E-02	2.0E-01	-0.26	2.4E-02	8.5E-02
	PC (32:1)	4.86_732.55	0.45	7.7E-05	**2.9E-03**	0.26	3.0E-02	9.2E-02
	PC (p-42:4) or PC (o-42:5)	6.56_850.67	-0.11	3.4E-01	5.5E-01	-0.22	2.9E-02	9.2E-02
	PC (35:2)	5.22_772.59	-0.16	1.6E-01	3.4E-01	-0.26	3.5E-02	1.0E-01
	PC (36:4) A	4.64_782.57	-0.13	2.7E-01	4.7E-01	-0.24	4.0E-02	1.1E-01
	PC (p-40:1) or PC (o-40:2)	6.64_828.68	-0.09	4.3E-01	6.2E-01	-0.23	4.3E-02	1.2E-01
	PC (40:5) B	5.80_836.61	0.26	2.4E-02	9.8E-02	0.22	5.9E-02	1.5E-01
	PC (34:2)	5.04_758.58	0.21	7.2E-02	1.9E-01	0.22	6.6E-02	1.5E-01
	PC (p-44:4) or PC (o-44:5)	7.17_878.70	-0.08	4.8E-01	6.7E-01	-0.2	6.6E-02	1.5E-01
	PC (33:0)	5.63_748.59	-0.01	9.4E-01	9.7E-01	-0.21	6.4E-02	1.5E-01
	PC (38:7)	4.20_804.55	-0.14	1.9E-01	3.8E-01	-0.18	8.1E-02	1.8E-01
	PC (32:0)	5.40_734.57	0.23	5.1E-02	1.5E-01	0.2	8.5E-02	1.9E-01
	PC (40:8)	4.41_830.57	-0.06	5.7E-01	7.4E-01	-0.18	9.9E-02	2.1E-01
	PC (p-44:5) or PC (o-44:6)	7.06_876.69	-0.18	9.6E-02	2.3E-01	-0.16	1.1E-01	2.3E-01
	PC (36:4) B	4.91_782.57	0.19	1.1E-01	2.5E-01	0.18	1.1E-01	2.3E-01
	PC (35:1)	5.80_774.61	-0.02	8.4E-01	9.2E-01	-0.18	1.1E-01	2.3E-01
	PC (35:4)	4.63_768.55	-0.03	7.7E-01	8.6E-01	-0.18	1.2E-01	2.4E-01
	PC (34:3)	4.60_756.56	0.15	2.2E-01	4.2E-01	0.17	1.4E-01	2.6E-01

	PC (40:6) A	5.04_834.60	-0.10	4.0E-01	5.9E-01	-0.17	1.4E-01	2.6E-01
	PC (p-36:1) or PC (o-36:2)	6.02_772.62	-0.11	4.0E-01	5.9E-01	-0.16	1.6E-01	2.9E-01
	PC (40:4)	5.98_838.63	0.26	2.1E-02	9.4E-02	0.16	1.8E-01	3.2E-01
	PC (p-40:3) or PC (o-40:4)	6.56_824.65	-0.14	2.2E-01	4.1E-01	-0.14	1.8E-01	3.2E-01
	PC (34:1)	5.52_760.59	0.33	5.5E-03	**4.2E-02**	0.15	1.9E-01	3.3E-01
	PC (37:4)	5.22_796.59	-0.19	1.2E-01	2.7E-01	-0.16	1.9E-01	3.3E-01
	PC (36:5) B	4.54_780.56	0.06	6.1E-01	7.6E-01	-0.14	1.9E-01	3.3E-01
	PC (31:0)	5.12_720.56	0.03	7.7E-01	8.6E-01	-0.15	1.9E-01	3.3E-01
	PC (p-42:5) or PC (o-42:6)	6.01_848.65	-0.16	1.8E-01	3.6E-01	-0.14	2.2E-01	3.6E-01
	PC (16:0/9:0(CHO))	2.38_650.44	0.08	4.0E-01	6.0E-01	-0.12	2.2E-01	3.6E-01
	PC (33:2)	4.70_744.55	-0.02	8.3E-01	9.2E-01	-0.14	2.3E-01	3.8E-01
	PC (36:6)	4.22_778.54	0.07	5.2E-01	7.0E-01	-0.12	2.4E-01	3.9E-01
	PC (p-34:0) or PC (o-34:1)	5.88_746.60	-0.12	3.0E-01	5.0E-01	-0.13	2.6E-01	4.2E-01
	PC (38:4) B	6.12_810.60	0.28	1.6E-02	7.7E-02	0.12	2.9E-01	4.4E-01
	PC (p-42:3) or PC (o-42:4)	7.21_852.69	-0.18	9.9E-02	2.3E-01	-0.11	2.8E-01	4.4E-01
	PC (p-32:0) or PC (o-32:1)	5.69_718.57	-0.03	7.7E-01	8.6E-01	-0.12	2.8E-01	4.4E-01
	PC (33:1)	5.16_746.57	0.14	2.5E-01	4.4E-01	-0.12	3.0E-01	4.6E-01
	PC (p-36:2) or PC (o-36:3)	5.48_770.61	0.04	7.2E-01	8.3E-01	-0.12	3.1E-01	4.6E-01
	PC (36:5) A	4.27_780.56	-0.05	6.6E-01	7.9E-01	-0.11	3.2E-01	4.8E-01
	PC (p-40:4) or PC (o-40:5)	5.92_822.64	-0.06	5.8E-01	7.5E-01	-0.1	3.4E-01	4.9E-01
	PC (28:0)	4.22_678.51	0.20	9.1E-02	2.2E-01	-0.11	3.4E-01	5.0E-01
	PC (40:5) A	5.57_8.36.61	0.08	4.6E-01	6.5E-01	-0.1	3.6E-01	5.0E-01
	PC (p-38:3) or PC (o-38:4)	5.91_796.62	-0.02	8.4E-01	9.2E-01	-0.1	3.5E-01	5.0E-01
	PC (37:3)	5.48_798.60	0.01	9.5E-01	9.7E-01	-0.11	3.6E-01	5.0E-01
	PC (p-36:4) or PC (o-36:5)	5.19_766.58	-0.02	8.5E-01	9.3E-01	-0.09	4.2E-01	5.6E-01
	PC (38:5) A	4.97_808.58	0.09	3.9E-01	5.9E-01	-0.09	4.3E-01	5.7E-01
	PC (p-38:4) or PC (o-38:5) B	5.80_794.61	0.01	9.0E-01	9.5E-01	-0.08	4.3E-01	5.7E-01
	PC (p-40:7) or PC (o-40:8)	5.09_816.57	-0.02	8.6E-01	9.3E-01	-0.08	4.5E-01	5.8E-01
	PC (p-34:1) or PC (o-34:2) A	5.36_744.59	0.02	8.9E-01	9.5E-01	-0.09	4.4E-01	5.8E-01
	PC (36:3) B	5.16_784.59	0.22	6.1E-02	1.7E-01	0.08	5.0E-01	6.3E-01
	PC (38:5) B	5.14_808.59	0.14	2.5E-01	4.4E-01	0.08	5.0E-01	6.3E-01
	PC (p-36:3) or PC (o-36:4)	5.28_768.59	0.15	2.4E-01	4.3E-01	0.08	5.1E-01	6.4E-01
	PC (36:3) A	5.07_784.59	-0.04	7.2E-01	8.3E-01	-0.08	5.1E-01	6.4E-01
	PC (42:5)	6.05_864.65	0.12	3.1E-01	5.1E-01	-0.07	5.4E-01	6.6E-01
	PC (36:1)	6.13_788.62	0.24	3.4E-02	1.2E-01	0.07	5.6E-01	6.8E-01
	PC (38:4) A	5.53_810.60	0.07	5.2E-01	7.1E-01	0.06	5.8E-01	6.9E-01
	PC (p-40:6) or PC (o-40:7) A	5.18_818.60	0.02	8.5E-01	9.2E-01	-0.06	5.8E-01	7.0E-01
	PC (36:2)	5.64_786.60	0.09	4.3E-01	6.2E-01	0.07	5.9E-01	7.1E-01
	PC (o-34:0)	6.44_748.62	0.04	7.5E-01	8.6E-01	0.06	6.0E-01	7.2E-01
	PC (o-32:0)	5.80_720.59	0.11	3.9E-01	5.9E-01	0.05	6.6E-01	7.6E-01
	PC (35:3)	4.86_770.57	0.05	6.3E-01	7.8E-01	-0.05	6.5E-01	7.6E-01
	PC (p-38:4) or PC (o-38:5) A	5.31_794.60	0.00	9.9E-01	9.9E-01	-0.05	6.6E-01	7.6E-01
	PC (38:2)	6.20_814.63	0.15	2.1E-01	4.1E-01	-0.05	6.9E-01	7.8E-01
	PC (34:0)	6.05_762.60	0.06	5.9E-01	7.5E-01	0.05	6.9E-01	7.8E-01
	PC (p-38:5) or PC (o-38:6)	5.12_792.59	0.03	8.2E-01	9.2E-01	-0.05	6.8E-01	7.8E-01
	PC (32:2)	4.43_730.54	0.20	9.2E-02	2.2E-01	0.04	7.1E-01	7.9E-01
	PC (36:4) C	5.48_782.57	0.26	2.4E-02	9.8E-02	0.04	7.2E-01	8.0E-01
	PC (p-40:6) or PC (o-40:7) B	5.60_818.60	0.01	9.4E-01	9.7E-01	-0.04	7.3E-01	8.1E-01
	PC (30:1)	4.33_704.52	0.36	2.1E-03	**2.2E-02**	0.04	7.5E-01	8.2E-01
	PC (42:6)	5.76_862.63	0.22	5.4E-02	1.6E-01	-0.04	7.4E-01	8.2E-01
	PC (p-38:2) or PC (o-38:3)	6.21_798.63	0.12	3.4E-01	5.5E-01	-0.02	8.7E-01	9.1E-01
	PC (32:3)	4.79_728.52	0.26	3.1E-02	1.1E-01	0.02	8.8E-01	9.2E-01
	PC (38:6) A	4.76_806.57	0.07	5.6E-01	7.3E-01	0.02	8.8E-01	9.2E-01
	PC (40:6) B	5.36_856.58	0.15	2.0E-01	3.8E-01	0.01	9.0E-01	9.3E-01

	PC (34:2)	4.98_780.55	0.07	5.3E-01	7.1E-01	-0.01	9.4E-01	9.6E-01
	PC (34:4)	4.37_754.54	0.19	8.6E-02	2.1E-01	0	9.7E-01	9.8E-01
	PC (30:0)	4.79_706.54	0.25	3.7E-02	1.3E-01	0	9.9E-01	9.9E-01
	PC (p-40:5) or PC (o-40:6)	5.71_820.62	-0.01	9.4E-01	9.7E-01	0	9.8E-01	9.9E-01
**Phosphatidylethanolamines (PE)**								
	PE (38:4)	5.70_768.56	0.20	9.5E-02	2.3E-01	0.4	7.5E-04	**7.3E-03**
	PE (36:1)	6.32_746.57	0.37	1.2E-03	**1.6E-02**	0.35	2.8E-03	**1.9E-02**
	PE (34:2)	5.17_716.53	0.26	2.6E-02	1.0E-01	0.32	5.0E-03	**2.8E-02**
	PE (p-36:4) or PE (o-36:5)	5.36_724.53	0.28	9.0E-03	5.8E-02	0.28	1.7E-02	6.7E-02
	PE (38:6)	4.91_764.52	0.13	2.4E-01	4.3E-01	0.22	5.4E-02	1.4E-01
	PE (p-38:5) or PE (o-38:6)	5.41_750.54	0.24	2.4E-02	9.8E-02	0.14	2.2E-01	3.6E-01
	PE (p-38:4) or PE (o-38:5)	6.01_752.56	0.16	1.4E-01	2.9E-01	0.1	3.6E-01	5.0E-01
	PE (p-40:5) or PE (o-40:6)	6.03_778.57	0.11	3.6E-01	5.6E-01	0.11	3.5E-01	5.0E-01
	PE (p-36:2) or PE (o-36:3)	6.13_728.56	0.07	5.3E-01	7.1E-01	-0.03	8.2E-01	8.7E-01
**Lysophosphatidylcholine (LPC)**								
	LPC (p-16:0) or LPC (o-16:1)	1.68_480.35	-0.20	4.1E-02	1.3E-01	-0.3	6.1E-03	**3.2E-02**
	LPC (15:0)	1.20_482.33	-0.05	6.6E-01	7.9E-01	-0.25	3.0E-02	9.2E-02
	LPC (18:1)	1.62_522.36	0.00	1.0E+00	1.0E+00	-0.21	6.3E-02	1.5E-01
	LPC (20:5)	0.96_542.32	-0.02	8.5E-01	9.3E-01	-0.16	1.1E-01	2.3E-01
	LPC (20:1)	2.37_550.39	-0.24	2.6E-02	1.0E-01	-0.17	1.3E-01	2.5E-01
	LPC (20:3)	1.42_546.36	0.24	2.0E-02	9.2E-02	0.17	1.5E-01	2.8E-01
	LPC (16:1)	1.09_494.32	0.23	4.7E-02	1.5E-01	0.15	1.9E-01	3.3E-01
	LPC (17:1)	1.35_508.34	0.03	8.0E-01	9.0E-01	-0.14	2.4E-01	3.9E-01
	LPC (18:0)	2.23_524.37	-0.14	1.9E-01	3.7E-01	-0.13	2.8E-01	4.4E-01
	LPC (18:2)	1.23_520.34	0.04	7.1E-01	8.3E-01	-0.1	3.4E-01	5.0E-01
	LPC (20:0)	3.05_552.40	-0.21	6.8E-02	1.8E-01	-0.1	3.8E-01	5.2E-01
	LPC (p-18:0) or LPC (o-18:1)	1.91_508.38	-0.15	1.7E-01	3.6E-01	-0.09	4.0E-01	5.5E-01
	LPC (22:6)	1.15_568.34	0.04	7.2E-01	8.3E-01	-0.08	4.4E-01	5.7E-01
	LPC (o-16:0)	1.75_482.36	-0.06	5.6E-01	7.3E-01	-0.08	4.7E-01	6.0E-01
	LPC (22:4)	1.66_572.37	0.10	3.6E-01	5.6E-01	0.08	5.2E-01	6.5E-01
	LPC (20:2)	1.79_548.37	-0.04	6.9E-01	8.1E-01	-0.07	5.6E-01	6.8E-01
	LPC (22:5)	1.30_570.36	-0.05	6.3E-01	7.8E-01	-0.06	6.1E-01	7.2E-01
	LPC (20:4)	1.20_544.34	0.01	9.0E-01	9.5E-01	-0.05	6.6E-01	7.6E-01
	LPC (14:0)	0.99_468.31	0.19	9.9E-02	2.3E-01	0.04	7.5E-01	8.2E-01
	LPC (18:3)	0.98_518.32	0.12	2.7E-01	4.7E-01	0.03	7.8E-01	8.4E-01
	LPC (16:0)	1.47_496.34	0.06	6.1E-01	7.6E-01	0.03	8.2E-01	8.7E-01
**Sphingolipids (SP)**								
**Ceramides**								
	Ceramide (d42:2)	7.59_670.61	0.16	1.9E-01	3.8E-01	0.22	5.0E-02	1.3E-01
	Ceramide (d42:2)	7.59_648.63	0.13	2.8E-01	4.7E-01	0.15	1.8E-01	3.3E-01
	Ceramide (d42:2)	7.59_630.62	0.07	5.9E-01	7.5E-01	0.14	2.1E-01	3.5E-01
	Ceramide (d42:1)	8.26_672.63	0.07	5.3E-01	7.1E-01	0.13	2.7E-01	4.2E-01
	Ceramide (d18:1/23:0)	7.94_636.63	0.12	3.0E-01	4.9E-01	0.13	2.9E-01	4.4E-01
	Ceramide (d42:1)	8.26_650.65	0.04	7.3E-01	8.4E-01	0.12	3.2E-01	4.8E-01
	Ceramide (d42:1)	8.26_632.63	0.05	6.9E-01	8.2E-01	0.11	3.3E-01	4.9E-01
	Ceramide (d40:1)	7.62_644.59	0.06	6.4E-01	7.8E-01	0.11	3.6E-01	5.0E-01
	Ceramide (d40:1)	7.62_622.61	0.05	6.5E-01	7.9E-01	0.11	3.5E-01	5.0E-01
	Ceramide (d18:1/23:0)	7.94_618.62	0.09	4.3E-01	6.2E-01	0.1	3.9E-01	5.4E-01
	Ceramide (d40:1)	7.62_604.60	0.06	6.2E-01	7.7E-01	0.1	4.2E-01	5.6E-01
	Ceramide (d18:1/23:0)	7.94_658.61	0.09	4.5E-01	6.4E-01	0.05	6.4E-01	7.5E-01
**Glucosylceramides**								
	Gal-Gal-Cer(d18:1/16:0) or Lactosylceramide(d18:1/16:0)	4.80_862.62	-0.22	5.2E-02	1.5E-01	-0.31	6.5E-03	**3.3E-02**

	Gal-Gal-Cer(d18:1/16:0) or Lactosylceramide(d18:1/16:0)	4.80_884.61	-0.22	4.9E-02	1.5E-01	-0.18	9.3E-02	2.0E-01
	Lactosylceramide (d18:1/24:1(15Z))	6.69_994.72	-0.15	2.0E-01	3.8E-01	-0.15	2.0E-01	3.4E-01
	Lactosylceramide (d18:1/24:1(15Z))	6.69_972.73	-0.10	3.6E-01	5.6E-01	-0.12	3.3E-01	4.9E-01
	GlcCer (d42:2)	6.96_792.67	0.14	2.5E-01	4.4E-01	0.2	8.5E-02	1.9E-01
	GlcCer (d42:1)	7.63_834.68	-0.14	2.3E-01	4.3E-01	-0.13	2.6E-01	4.1E-01
	GlcCer (d40:1)	6.98_784.67	-0.09	4.5E-01	6.4E-01	-0.13	2.6E-01	4.2E-01
	GlcCer (d40:1)	6.98_806.65	-0.14	2.5E-01	4.4E-01	-0.12	2.9E-01	4.4E-01
	GlcCer (d42:1)	7.63_812.70	-0.14	2.6E-01	4.5E-01	-0.11	3.2E-01	4.8E-01
	GlcCer (d42:2)	6.96_832.66	-0.11	3.8E-01	5.8E-01	-0.1	4.1E-01	5.5E-01
	GlcCer (d42:1)	7.63_794.69	-0.07	5.6E-01	7.3E-01	-0.09	4.5E-01	5.8E-01
	GlcCer (d40:1)	6.98_766.66	-0.09	4.3E-01	6.2E-01	-0.07	5.3E-01	6.5E-01
	GlcCer (d42:2)	6.96_810.68	-0.05	6.7E-01	8.0E-01	-0.05	6.6E-01	7.6E-01
**Sphingomyelins (SM)**								
	SM (d36:0)	5.61_733.62	0.29	8.1E-03	5.6E-02	0.38	3.7E-04	**4.9E-03**
	SM (d38:2)	5.46_757.62	-0.24	5.3E-02	1.5E-01	-0.35	2.7E-03	**1.9E-02**
	SM (d39:2)	5.82_771.64	-0.22	6.9E-02	1.8E-01	-0.36	3.0E-03	**2.0E-02**
	SM (d40:0)	6.96_789.68	0.29	1.2E-02	6.5E-02	0.3	6.4E-03	**3.2E-02**
	SM (d41:2)	6.34_799.67	-0.14	2.1E-01	4.1E-01	-0.31	8.0E-03	**3.8E-02**
	SM (d30:1)	3.63_647.51	-0.17	1.4E-01	2.9E-01	-0.28	1.3E-02	5.3E-02
	SM (d43:2)	6.99_827.70	-0.14	2.3E-01	4.2E-01	-0.28	2.1E-02	7.8E-02
	SM (d37:1)	5.72_745.62	-0.17	1.5E-01	3.2E-01	-0.27	2.5E-02	8.7E-02
	SM (d32:2)	3.72_673.53	-0.16	1.7E-01	3.5E-01	-0.26	2.9E-02	9.1E-02
	SM (d38:0)	6.29_761.65	0.22	6.7E-02	1.8E-01	0.23	3.1E-02	9.5E-02
	SM (d42:2)	6.79_813.69	-0.02	9.1E-01	9.6E-01	-0.29	3.1E-02	9.5E-02
	SM (d43:1)	7.55_829.71	-0.19	1.1E-01	2.5E-01	-0.23	4.5E-02	1.2E-01
	SM (d39:1)	6.37_773.65	-0.10	3.6E-01	5.6E-01	-0.24	4.1E-02	1.2E-01
	SM (d40:2)	6.12_785.66	-0.21	9.2E-02	2.2E-01	-0.24	4.6E-02	1.3E-01
	SM (d43:2)	6.88_827.70	-0.19	1.0E-01	2.4E-01	-0.23	4.6E-02	1.3E-01
	1_SM 17:0	5.06_717.59	-0.06	5.6E-01	7.3E-01	-0.2	5.1E-02	1.3E-01
	SM (d33:1)	4.42_689.56	-0.11	3.6E-01	5.6E-01	-0.23	5.5E-02	1.4E-01
	SM (d32:1)	4.13_675.54	-0.10	4.0E-01	5.9E-01	-0.21	6.9E-02	1.6E-01
	SM (d38:1)	6.02_759.64	-0.08	5.0E-01	6.9E-01	-0.18	1.2E-01	2.4E-01
	SM (d42:3)	6.11_811.67	-0.06	6.1E-01	7.6E-01	-0.16	1.6E-01	3.0E-01
	SM (d32:0)	4.35_677.56	-0.05	6.7E-01	8.0E-01	-0.15	1.8E-01	3.2E-01
	SM (d40:2)	6.01_785.66	-0.02	8.9E-01	9.5E-01	-0.15	2.0E-01	3.4E-01
	SM (d42:0)	7.61_817.72	0.15	2.1E-01	4.1E-01	0.14	2.1E-01	3.5E-01
	SM (d36:1)	5.35_731.61	0.05	6.5E-01	7.9E-01	0.09	4.4E-01	5.7E-01
	SM (d36:3)	4.38_727.57	-0.10	3.7E-01	5.6E-01	-0.08	5.0E-01	6.3E-01
	SM (d34:2)	4.23_701.56	0.02	8.9E-01	9.5E-01	-0.08	5.2E-01	6.5E-01
	SM (d34:1)	4.72_703.58	0.00	9.7E-01	9.8E-01	-0.06	6.1E-01	7.2E-01
	SM (d42:2)	6.67_813.69	0.11	3.8E-01	5.8E-01	0.05	6.6E-01	7.6E-01
	SM (d42:1)	7.35_815.70	0.04	7.6E-01	8.6E-01	-0.05	6.6E-01	7.6E-01
	SM (d41:1)	6.90_801.68	0.08	5.2E-01	7.0E-01	-0.05	6.9E-01	7.8E-01
	SM (d44:2)	7.31_841.72	-0.01	9.5E-01	9.7E-01	-0.04	7.0E-01	7.9E-01
	SM (d36:2)	4.82_729.59	-0.01	9.1E-01	9.6E-01	0.01	9.2E-01	9.5E-01
	SM (d40:1)	6.69_787.67	0.04	7.3E-01	8.4E-01	0.01	9.6E-01	9.8E-01
	SM (d34:0)	4.94_727.57	-0.01	9.3E-01	9.7E-01	-0.01	9.6E-01	9.8E-01
**Sterol Lipids**								
**Cholesteryl esters (CE)**								
	CE (18:1)	10.85_673.59	-0.40	9.0E-04	**1.3E-02**	-0.53	9.8E-07	**4.1E-04**
	CE (18:1)	10.85_668.63	-0.36	2.8E-03	**2.7E-02**	-0.4	2.6E-04	**4.7E-03**
	CE (20:4)	10.13_695.57	-0.31	8.0E-03	5.6E-02	-0.38	8.4E-04	**7.7E-03**
	CE (20:5)	9.71_693.56	-0.24	4.9E-02	1.5E-01	-0.34	2.4E-03	**1.7E-02**

	CE (22:6)	9.88_719.57	-0.34	3.8E-03	**3.3E-02**	-0.34	3.9E-03	**2.5E-02**
	CE (18:2)	10.37_671.57	-0.52	1.2E-05	**1.1E-03**	-0.33	5.2E-03	**2.9E-02**
	CE (18:3)	9.96_669.56	-0.35	4.1E-03	**3.4E-02**	-0.28	1.7E-02	6.7E-02
	CE (20:3)	10.49_692.63	-0.24	4.4E-02	1.4E-01	-0.27	1.7E-02	6.7E-02
	CE (20:4)	10.13_690.62	-0.20	8.7E-02	2.1E-01	-0.26	2.4E-02	8.5E-02
	CE (20:3)	10.57_697.59	-0.28	1.8E-02	8.5E-02	-0.27	2.5E-02	8.8E-02
	CE (20:5)	9.70_688.60	-0.16	1.6E-01	3.4E-01	-0.24	2.8E-02	9.1E-02
	CE (18:2)	10.37_666.62	-0.48	8.8E-05	**2.9E-03**	-0.26	3.4E-02	1.0E-01
	CE (22:6)	9.88_714.62	-0.13	2.8E-01	4.7E-01	-0.19	1.0E-01	2.2E-01
	CE (16:1)	10.34_640.60	0.28	1.8E-02	8.5E-02	0.16	1.6E-01	3.0E-01
	CE (18:3)	9.96_664.60	-0.11	3.9E-01	5.9E-01	-0.17	1.8E-01	3.2E-01
	CE (16:1)	10.33_645.56	0.16	1.8E-01	3.7E-01	-0.01	9.4E-01	9.6E-01
**Cholesterol**								
	Cholesterol	4.87_369.35	0.06	5.9E-01	7.5E-01	0.12	3.4E-01	5.0E-01
**Unannotated**								
	CSH_posESI #176	10.80_551.50	0.50	1.6E-05	**1.1E-03**	0.51	3.6E-06	**6.4E-04**
	CSH_posESI #022	6.39_615.49	0.36	1.6E-03	**2.0E-02**	0.47	3.0E-05	**1.6E-03**
	CSH_posESI #282	10.80_577.52	0.42	2.2E-04	**5.4E-03**	0.44	4.5E-05	**2.1E-03**
	CSH_posESI #081	11.17_951.78	0.21	7.7E-02	2.0E-01	0.41	1.3E-04	**3.7E-03**
	CSH_posESI #121	11.55_933.86	0.30	1.3E-02	6.9E-02	0.44	2.0E-04	**4.6E-03**
	CSH_posESI #141	11.20_899.75	0.40	3.6E-04	**6.4E-03**	0.4	2.9E-04	**4.7E-03**
	CSH_posESI #058	10.31_951.81	0.37	1.7E-03	**2.0E-02**	0.39	6.5E-04	**7.1E-03**
	CSH_posESI #298	10.82_603.53	0.25	3.5E-02	1.2E-01	0.36	1.1E-03	**9.5E-03**
	CSH_posESI #017	10.39_549.49	0.47	3.0E-05	**1.6E-03**	0.37	1.1E-03	**9.7E-03**
	CSH_posESI #187	10.46_601.52	0.21	6.6E-02	1.8E-01	0.34	1.4E-03	**1.2E-02**
	CSH_posESI #214	11.20_931.85	0.22	7.9E-02	2.0E-01	0.35	2.5E-03	**1.8E-02**
	CSH_posESI #079	11.55_959.88	0.18	1.3E-01	2.8E-01	0.33	3.8E-03	**2.5E-02**
	CSH_posESI #006	10.17_896.79	0.32	5.4E-03	**4.1E-02**	0.33	4.7E-03	**2.7E-02**
	CSH_posESI #364	7.27_854.70	-0.29	8.4E-03	5.6E-02	-0.31	6.1E-03	**3.2E-02**
	CSH_posESI #359	10.86_663.45	-0.28	1.3E-02	6.9E-02	-0.32	7.4E-03	**3.5E-02**
	CSH_posESI #004	5.42_780.61	-0.21	7.4E-02	1.9E-01	-0.29	8.6E-03	**3.9E-02**
	CSH_posESI #109	10.88_929.83	0.10	4.3E-01	6.2E-01	0.3	9.6E-03	**4.2E-02**
	CSH_posESI #116	9.36_885.68	0.31	9.9E-03	6.1E-02	0.3	1.7E-02	6.7E-02
	CSH_posESI #310	10.39_1017.94	-0.44	3.6E-04	**6.4E-03**	-0.27	2.4E-02	8.5E-02
	CSH_posESI #103	4.86_766.57	-0.01	9.5E-01	9.7E-01	-0.26	2.9E-02	9.1E-02
	CSH_posESI #031	9.72_958.81	0.26	2.8E-02	1.0E-01	0.25	3.4E-02	1.0E-01
	CSH_posESI #261	5.04_1516.13	0.13	2.9E-01	4.9E-01	0.25	4.0E-02	1.1E-01
	CSH_posESI #170	10.01_867.69	0.19	6.2E-02	1.7E-01	0.22	4.9E-02	1.3E-01
	CSH_posESI #033	4.63_872.84	-0.14	2.5E-01	4.4E-01	-0.23	4.7E-02	1.3E-01
	CSH_posESI #201	10.38_369.35	-0.49	4.9E-05	**2.0E-03**	-0.23	6.0E-02	1.5E-01
	CSH_posESI #164	9.95_841.67	0.36	2.6E-03	**2.5E-02**	0.22	6.2E-02	1.5E-01
	CSH_posESI #215	10.15_808.74	0.29	1.4E-02	7.2E-02	0.2	7.2E-02	1.7E-01
	CSH_posESI #062	5.52_1542.18	0.30	1.2E-02	6.8E-02	0.2	9.0E-02	2.0E-01
	CSH_posESI #192	9.80_973.80	0.06	6.3E-01	7.8E-01	0.19	9.4E-02	2.1E-01
	CSH_posESI #046	5.52_1542.64	0.31	1.0E-02	6.1E-02	0.19	1.1E-01	2.3E-01
	CSH_posESI #197	6.70_876.69	-0.26	2.7E-02	1.0E-01	-0.18	1.1E-01	2.3E-01
	CSH_posESI #025	9.62_970.79	0.01	9.6E-01	9.7E-01	0.19	1.1E-01	2.3E-01
	CSH_posESI #117	10.66_915.81	0.21	8.2E-02	2.1E-01	0.19	1.2E-01	2.4E-01
	CSH_posESI #203	11.20_925.76	-0.12	2.6E-01	4.4E-01	-0.18	1.2E-01	2.4E-01
	CSH_posESI #232	5.52_1162.85	0.26	2.3E-02	9.8E-02	0.18	1.3E-01	2.5E-01
	CSH_posESI #104	8.97_764.68	0.22	7.6E-02	1.9E-01	0.18	1.4E-01	2.6E-01
	CSH_posESI #205	3.15_381.30	-0.05	6.1E-01	7.6E-01	-0.14	1.4E-01	2.6E-01
	CSH_posESI #125	9.62_865.67	0.08	5.1E-01	7.0E-01	0.17	1.5E-01	2.8E-01
	CSH_posESI #098	9.50_813.64	0.26	3.4E-02	1.2E-01	0.16	1.8E-01	3.2E-01
	CSH_posESI #057	8.90_738.66	0.22	6.5E-02	1.8E-01	0.15	2.0E-01	3.4E-01
	CSH_posESI #288	4.87_577.52	0.27	1.6E-02	7.7E-02	0.14	2.1E-01	3.5E-01
	CSH_posESI #091	6.97_902.70	-0.06	5.6E-01	7.3E-01	-0.12	2.4E-01	3.9E-01
	CSH_posESI #336	10.37_663.45	-0.27	2.4E-02	9.8E-02	-0.14	2.5E-01	4.0E-01
	CSH_posESI #124	5.19_864.53	0.23	4.3E-02	1.4E-01	0.12	2.9E-01	4.4E-01

	CSH_posESI #037	5.57_858.60	0.04	7.3E-01	8.4E-01	-0.12	2.9E-01	4.4E-01
	CSH_posESI #056	8.25_694.68	0.09	4.1E-01	6.1E-01	0.12	3.0E-01	4.6E-01
	CSH_posESI #363	5.53_792.57	0.07	5.5E-01	7.3E-01	-0.12	3.2E-01	4.8E-01
	CSH_posESI #127	10.07_960.82	-0.04	7.0E-01	8.2E-01	0.1	3.3E-01	4.9E-01
	CSH_posESI #137	9.12_816.70	0.20	8.9E-02	2.2E-01	0.11	3.4E-01	5.0E-01
	CSH_posESI #043	5.66_893.56	-0.06	5.9E-01	7.5E-01	-0.11	3.5E-01	5.0E-01
	CSH_posESI #165	5.58_1583.13	0.06	6.0E-01	7.6E-01	0.09	3.6E-01	5.0E-01
	CSH_posESI #106	5.26_790.58	0.16	1.9E-01	3.7E-01	0.1	3.8E-01	5.3E-01
	CSH_posESI #296	4.97_603.53	-0.12	3.3E-01	5.4E-01	0.1	4.0E-01	5.5E-01
	CSH_posESI #290	5.64_866.54	0.06	4.8E-01	6.7E-01	-0.07	4.0E-01	5.5E-01
	CSH_posESI #122	5.06_1475.16	0.06	5.8E-01	7.5E-01	-0.09	4.1E-01	5.5E-01
	CSH_posESI #303	5.19_874.55	0.14	1.4E-01	2.9E-01	0.08	4.2E-01	5.6E-01
	CSH_posESI #155	5.62_824.57	0.09	2.3E-01	4.2E-01	0.06	4.2E-01	5.6E-01
	CSH_posESI #307	4.95_872.54	-0.09	3.4E-01	5.4E-01	-0.08	4.2E-01	5.6E-01
	CSH_posESI #100	5.04_1567.59	0.01	9.1E-01	9.6E-01	0.09	4.3E-01	5.7E-01
	CSH_posESI #061	5.22_748.54	0.20	4.7E-02	1.5E-01	0.08	4.5E-01	5.8E-01
	CSH_posESI #293	4.81_776.58	0.09	4.3E-01	6.2E-01	0.09	4.5E-01	5.8E-01
	CSH_posESI #168	5.09_1172.86	-0.01	9.4E-01	9.7E-01	-0.09	4.5E-01	5.8E-01
	CSH_posESI #304	5.55_1533.11	0.32	1.3E-03	**1.6E-02**	0.07	5.2E-01	6.4E-01
	CSH_posESI #003	4.82_805.62	0.09	5.6E-01	7.3E-01	-0.09	5.2E-01	6.5E-01
	CSH_posESI #015	3.44_279.16	0.03	7.4E-01	8.4E-01	-0.05	5.4E-01	6.6E-01
	CSH_posESI #036	4.85_858.60	-0.01	9.6E-01	9.7E-01	0.08	5.4E-01	6.6E-01
	CSH_posESI #159	5.53_832.58	0.00	9.7E-01	9.8E-01	0.07	5.4E-01	6.6E-01
	CSH_posESI #211	10.06_893.70	-0.28	1.3E-02	6.9E-02	0.06	6.2E-01	7.3E-01
	CSH_posESI #285	5.62_876.57	-0.06	3.5E-01	5.5E-01	-0.04	6.1E-01	7.3E-01
	CSH_posESI #308	6.18_878.59	0.25	2.4E-02	9.8E-02	0.05	6.4E-01	7.5E-01
	CSH_posESI #301	5.51_850.56	0.11	2.2E-01	4.2E-01	0.04	6.4E-01	7.5E-01
	CSH_posESI #230	5.06_848.54	0.05	6.1E-01	7.6E-01	0.05	6.9E-01	7.8E-01
	CSH_posESI #001	4.02_752.59	0.01	9.6E-01	9.7E-01	-0.05	6.9E-01	7.8E-01
	CSH_posESI #284	5.63_1201.85	0.11	3.7E-01	5.6E-01	0.05	7.0E-01	7.9E-01
	CSH_posESI #268	5.62_1573.19	0.10	4.0E-01	5.9E-01	0.04	7.3E-01	8.1E-01
	CSH_posESI #156	3.45_803.55	0.04	6.5E-01	7.9E-01	0.03	7.3E-01	8.1E-01
	CSH_posESI #002	4.55_719.57	0.05	6.9E-01	8.1E-01	0.04	7.3E-01	8.1E-01
	CSH_posESI #040	5.03_865.53	0.09	4.1E-01	6.1E-01	0.04	7.5E-01	8.2E-01
	CSH_posESI #212	5.04_1538.62	0.08	5.0E-01	6.9E-01	0.04	7.4E-01	8.2E-01
	CSH_posESI #041	5.22_430.38	-0.12	2.4E-01	4.3E-01	-0.03	7.7E-01	8.4E-01
	CSH_posESI #231	5.06_838.51	0.07	5.4E-01	7.2E-01	0.03	7.7E-01	8.4E-01
	CSH_posESI #270	4.92_627.53	-0.02	8.8E-01	9.5E-01	-0.03	7.8E-01	8.4E-01
	CSH_posESI #362	5.47_867.54	-0.08	4.4E-01	6.3E-01	0.03	7.9E-01	8.5E-01
	CSH_posESI #134	10.45_895.72	0.01	9.3E-01	9.7E-01	0.02	7.9E-01	8.5E-01
	CSH_posESI #042	5.56_900.57	-0.11	3.2E-01	5.2E-01	0.03	8.1E-01	8.6E-01
	CSH_posESI #130	6.67_903.66	0.03	7.8E-01	8.8E-01	-0.02	8.2E-01	8.7E-01
	CSH_posESI #027	3.44_391.29	0.02	8.4E-01	9.2E-01	-0.01	8.4E-01	8.9E-01
	CSH_posESI #142	3.45_435.32	0.01	9.6E-01	9.7E-01	-0.01	8.7E-01	9.1E-01
	CSH_posESI #275	4.92_887.56	-0.02	8.9E-01	9.5E-01	-0.02	8.9E-01	9.2E-01
	CSH_posESI #034	4.76_896.54	-0.04	7.2E-01	8.3E-01	0.01	9.3E-01	9.6E-01
	CSH_posESI #286	5.04_1159.84	0.06	6.2E-01	7.7E-01	0	9.8E-01	9.8E-01
	CSH_posESI #196	10.80_897.73	0.02	8.9E-01	9.5E-01	0	9.8E-01	9.8E-01

Linear mixed models adjusted for gestational age at blood collection, maternal age, enrollment BMI, race/ethnicity, alcohol use before pregnancy, and family history of diabetes.
FDR q-values <0.05 are bold faced
