# Supplementary figures and images for: Plasma lipidomics profile in pregnancy and gestational diabetes risk: a prospective study in a multiracial/ethnic cohort
Source: BMJ Open Diabetes Res Care. 2021 Mar 5;9(1):e001551. doi: 10.1136/bmjdrc-2020-001551 (PMC7939004; doi:10.1136/bmjdrc-2020-001551)

**A) Visit 0 (10-14 weeks)**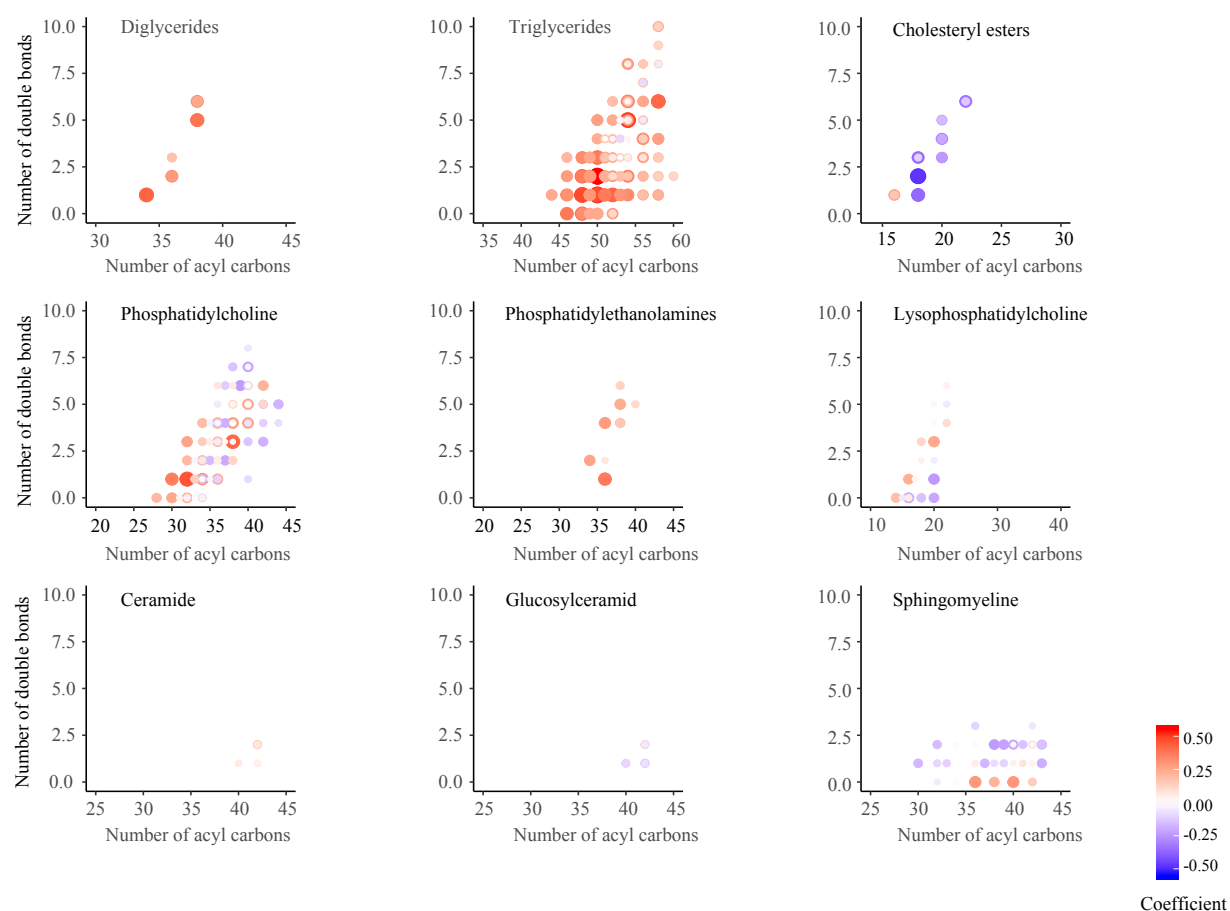**B) Visit 1 (15-26 weeks)**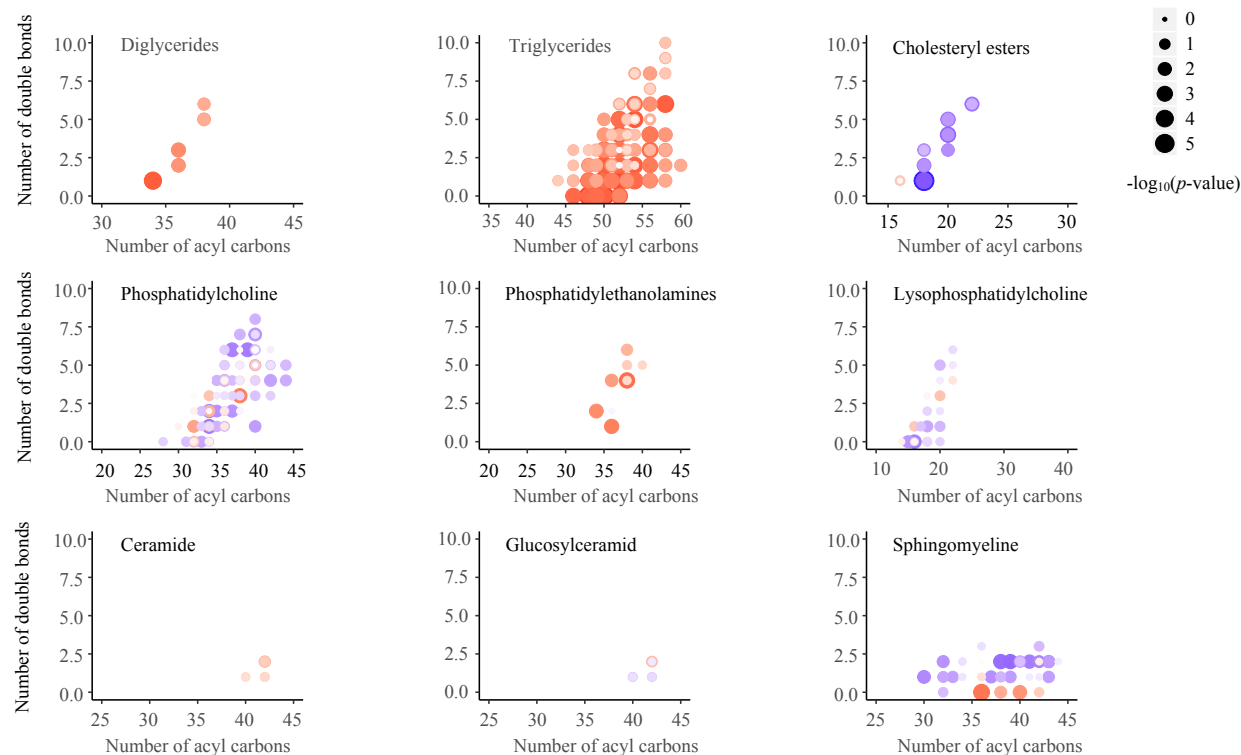

Supplement: Supplementary data [file bmjdrc-2020-001551supp004.pdf]

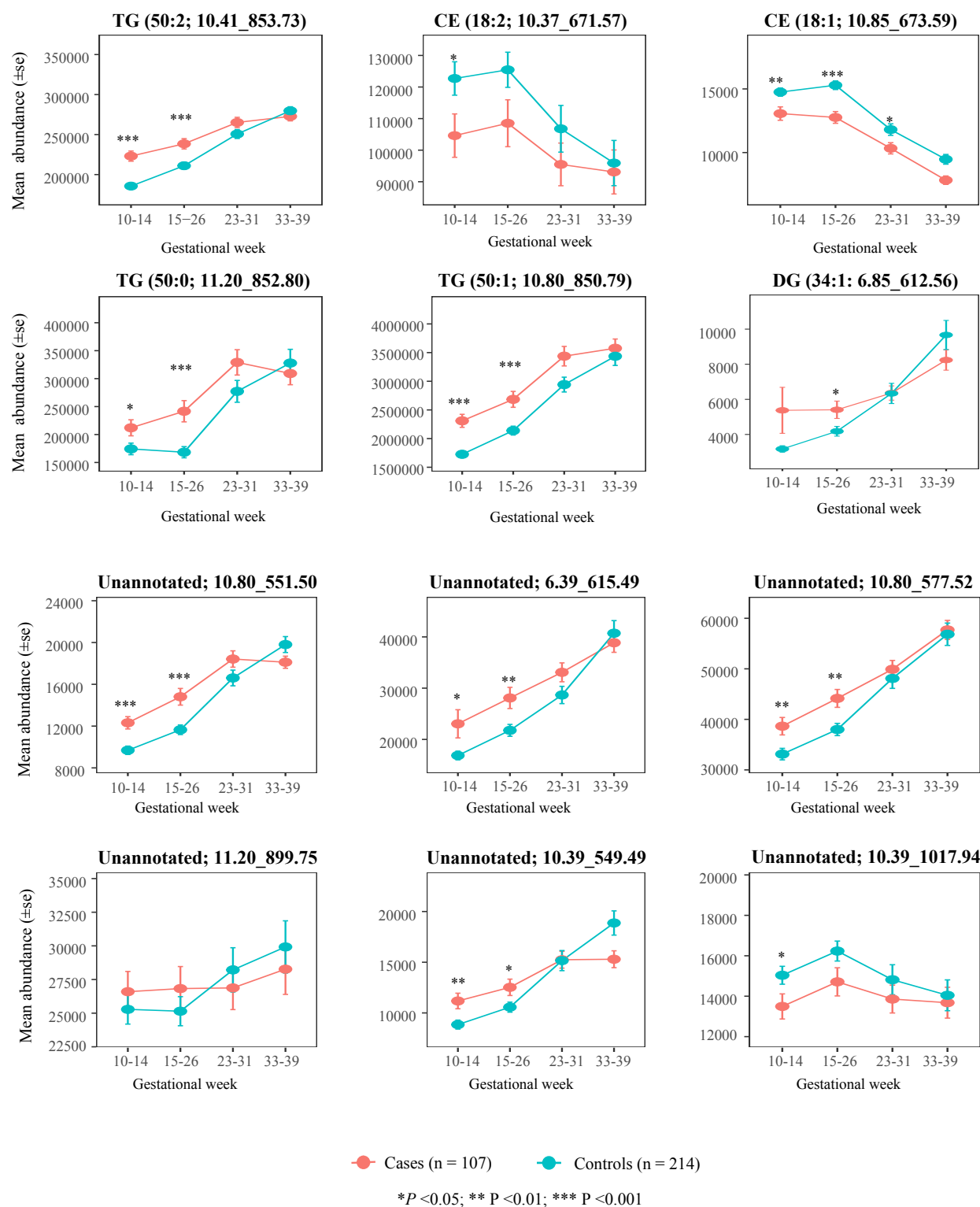

Supplement: Supplementary data [file bmjdrc-2020-001551supp005.pdf]
