# Supplementary material for: Plasma lipidomics profile in pregnancy and gestational diabetes risk: a prospective study in a multiracial/ethnic cohort
Source: BMJ Open Diabetes Res Care. 2021 Mar 5;9(1):e001551. doi: 10.1136/bmjdrc-2020-001551 (PMC7939004; doi:10.1136/bmjdrc-2020-001551)
Supplement: Supplementary data [file bmjdrc-2020-001551supp006.pdf]

Supplementary table S2. Associations of lipid networks with GDM risk stratified by disease severity and study visit

Lipid networks	Visit 0 (10-14 weeks)						Visit 1 (15-26 weeks)					
	GDM women not taking medication and their matched controls (n= 144; 45 cases, 99 controls)			GDM Women taking medication and their matched controls (n = 144; 48 cases and 96 controls)			GDM women not taking medication and their matched controls (n= 140; 47 cases, 93 controls)			GDM Women taking medication and their matched controls (n = 155; 52 cases and 103 controls)		
	β	se	*p*-value	β	se	*p*-value	β	se	*p*-value	β	se	*p*-value
Red	0.000	0.009	0.988	-0.011	0.009	0.230	0.006	0.018	0.742	0.050	0.032	0.121
Turquoise	-0.024	0.007	**0.001**	-0.006	0.007	0.417	-0.028	0.013	**0.038**	-0.016	0.021	0.438
Yellow	0.030	0.010	**0.005**	0.013	0.011	0.252	0.024	0.017	0.178	0.021	0.028	0.460
Black	-0.018	0.008	**0.039**	0.000	0.007	0.965	-----	-----	-----	-----	-----	-----
Blue	-0.021	0.007	**0.002**	0.001	0.007	0.940	-0.039	0.015	**0.010**	0.000	0.024	0.997
Brown	0.027	0.008	**0.001**	0.006	0.009	0.469	0.033	0.024	0.176	0.025	0.036	0.488
Green	-0.008	0.010	0.434	-0.002	0.011	0.889	0.001	0.022	0.980	-0.032	0.040	0.429
Pink	0.014	0.009	0.111	0.004	0.010	0.673	-----	-----	-----	-----	-----	-----

Models were adjusted for maternal age, enrollment BMI, family history of diabetes, alcohol use before pregnancy, race/ethnicity, and gestational age at blood collection
